# Triptolide Inhibits Epileptic Seizures by Rescuing the Neuroinflammation‐Related GABAergic Dysfunction in Mice

**DOI:** 10.1111/cns.70586

**Published:** 2025-08-29

**Authors:** Haimei Lu, Yijun Luo, Mingxuan Han, Yitian Lan, Wenmi Li, Xiu Yu, Xiaoyu Wang, Rongrong Chen, Huawei Zhao, Zhenghao Xu

**Affiliations:** ^1^ Research Institute of Chinese Medical Clinical Foundation and Immunology & TCM Science and Research Center, Wenzhou TCM Hospital of Zhejiang Chinese Medical University, College of Basic Medical Science Zhejiang Chinese Medical University Zhejiang China; ^2^ General Surgery Department, Children's Hospital Zhejiang University School of Medicine and National Clinical Research Center for Child Health Hangzhou Zhejiang China; ^3^ Department of Pharmacy, Children's Hospital Zhejiang University School of Medicine and National Clinical Research Center for Child Health Hangzhou Zhejiang China

**Keywords:** IL‐1β, neural circuit dysfunction, neuroinflammation, temporal lobe epilepsy, triptolide

## Abstract

**Objective:**

This study aims to evaluate the antiepileptic effect of triptolide (TPL), a strong anti‐inflammatory and immunosuppressive diterpenoid compound from a Chinese herb medicine Tripterygium wilfordii Hook F (TWHF).

**Methods:**

The pentylenetetrazol (PTZ)‐induced seizure model, maximal electroshock seizure (MES) model, corneal (6 Hz) kindling model, and kainic acid (KA) mouse model were used to assess the antiepileptic effect of TPL. EEG recording and behavioral tests were used to evaluate the disease‐modifying effects of TPL in epileptic conditions. Extracellular recording combined with optogenetics was used to evaluate the effect of TPL on hippocampal GABAergic inhibition in vivo.

**Results:**

Though intragastric administration of TPL (50 μg/kg) had no direct anticonvulsive effect in normal mice, it retarded corneal kindling acquisition in mice and reduced the seizure severity in corneal kindled mice. Meanwhile, intragastric administration (10 or 50 μg/kg) of TPL exhibited disease‐modifying effects in the established KA mouse model of temporal lobe epilepsy (TLE). Extracellular recording combined with optogenetics showed that TPL improved the hippocampal GABAergic “braking effect” in epileptic mice. Furthermore, bioinformatics analysis predicts IL‐1β as a key target for the antiepileptic effect of TPL. TPL reduced the level of IL‐1β in the hippocampus of KA‐induced epileptic mice, while focal injection of IL‐1β (5 ng in 1 μL saline) attenuated the hippocampal GABAergic “braking effect” in normal mice. Focal injection of TPCA‐1 (an IKK‐2/NF‐κB inhibitor, 600 ng in 1 μL 1% DMSO) partly recovered the IL‐1β‐induced attenuation of the GABAergic “braking effect”.

**Conclusion:**

This study indicates that a low dose of TPL may inhibit epilepsy by potentially rescuing dysfunction in hippocampal GABAergic neural circuits related to neuroinflammation in mice.

## Graphical abstract

Triptolide suppresses the expression of cytokines such as IL‐β, thereby ameliorating neuroinflammation‐related GABAergic dysfunction and inhibiting epileptic seizure in mice.

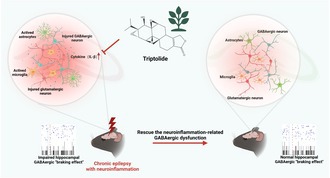

## 1 Introduction

Epilepsy is one of the most common types of neural disorder in adults, which is characterized by spontaneous recurring seizures. Despite classical and newly developed treatment approaches, such as antiseizure medications (ASMs) therapy, surgery, and brain stimulation, about one‐third of patients continue to have seizures [1, 2]. Recurrent uncontrolled epileptic seizures may result in accident injuries, cognitive impairments, and unexpected morbidity [3]. Therefore, it remains essential to develop drugs for the control of epileptic seizures.

Brain inflammation is one major factor in epilepsy [4, 5, 6]. Brain inflammation may occur either during epileptogenesis or after chronic spontaneous recurring seizures [7, 8, 9]. Brain inflammation in epilepsy is primarily characterized by the proinflammatory mediators in the epileptic focus [10], such as interleukin‐1β (IL‐1β) [11, 12], high‐mobility group box 1 (HMGB1) [13, 14, 15], and tumor necrosis factor‐α (TNF‐α) [16, 17]. These upregulated proinflammatory mediators may lead to neural excitability, promote epileptogenesis, and aggravate seizure severity. Thus, targeting brain inflammation has been considered as a promising way for the treatment of epilepsy.

Triptolide (TPL) is a strong anti‐inflammatory and immunosuppressive compound from a Chinese herb Tripterygium wilfordii Hook F (TWHF) [18, 19]. TPL is the main active ingredient of Tripterygium Glycosides Tablets (such as Approval No. Z33020422) and Tripterygium Wilfordii Tablets (such as Approval No. Z42021534) that are approved by the China Food and Drug Administration (http://samr.cfda.gov.cn). TWHF is relatively safe at low dosage and has been widely used for autoimmune diseases in China, such as rheumatoid arthritis [20]. Its chief component, TPL, can easily access the brain and may be of benefit to some neurologic disorders, such as Alzheimer disease, depression, and neuropathic pain [21, 22, 23].

In the present study, we found TPL acted antiepileptic effect in a classic kainic acid (KA) induced chronic mouse model of temporal lobe epilepsy (TLE), at least in part, by inhibiting neuro‐inflammation and improving the “braking effect” of the GABAergic system in the dentate gyrus (DG) region. Importantly, we further found that direct intrahippocampal administration of IL‐1β acutely impaired of the “braking effect” of DG GABAergic system in normal mice.

## 2 Materials and Methods

### 2.1 Animals

C57BL/6 mice and vesicular GABA transporter (VGAT)‐Channelrhodopsin 2 (ChR2)‐enhanced Yellow Fluorescent Protein (eYFP) mice (VGAT‐ChR2‐eYFP mice; No. 014548) were reared as previously described and were genotyped according to the protocols promulgated by the Jackson Laboratory. All mice used in this study were male and were 2 to 4 months old. Mice were group‐housed with a 12 h/12 h light–dark cycle (lights on from 8:00 to 20:00). All animals received water and food *ad libitum*. The use and care of the mice were in accordance with the guidelines of the Animal Advisory Committee of Zhejiang Chinese Medical University and the US National Institutes of Health Guidelines for the Care and Use of Laboratory Animals. This study was approved by the Institutional Animal Care and Use Committee of Zhejiang Chinese Medical University.

### 2.2 Chemicals

Triptolide (TPL, Shanghai Yuanye, China, B20709) was dissolved in dimethyl sulfoxide (DMSO) to obtain a 1 mg/mL stock solution. The stock solution was stored at −20°C and further diluted with water to the concentration of administration before use. The doses of 1, 10, and 50 μg/kg were administered to evaluate the antiepileptic effects of TPL, with the optimal dose of 50 μg/kg being selected for subsequent mechanistic investigations. These triptolide doses were selected based on the following rationale: (1) Similar doses have demonstrated biological activity in animal models of neurologic disorders [24, 25, 26]; (2) This dosing strategy represents a clinically relevant low dose when compared to therapeutic regimens of Tripterygium wilfordii extracts in autoimmune diseases; (3) This dosing strategy was designed to maintain pharmacological efficacy while minimizing potential adverse effects.

Pentylenetetrazol (PTZ, Sigama, P6500) and kainic acid (KA, Sigma, K0250) were respectively dissolved in saline at the concentration required for injection.

Human recombinant IL‐1β was dissolved in saline at 5 ng/μL. Interleukin‐1 receptor antagonist (IL‐1RA) was dissolved in saline at 100 ng/μL. TPCA‐1 (an IKK‐2/NF‐κB inhibitor) was dissolved in DMSO to obtain a 60 mg/mL stock solution, and further diluted with saline to 600 ng/μL of administration before use.

### 2.3 Drug Administration

1. To evaluate the antiepileptic effect of TPL in the acute seizure model, normal mice were administered water or TPL (50 μg/kg) for 2 weeks prior to the experiment.
2. To evaluate the effects of TPL on corneal kindling acquisition, mice were administered water or TPL (50 μg/kg) once per day, 30 min prior to the first kindling stimulation.
3. To evaluate the effects of TPL on corneal kindled mice, the mice were administered water or TPL (50 μg/kg) once per day for 10 days, 30 min prior to the kindling stimulation.
4. To evaluate the antiepileptic effects of TPL in the KA model, mice were administered water or TPL (50 μg/kg) once per day for 2 weeks after modeling was completed (28 days after KA injection). Notably, dosages of 1 and 10 μg/kg were exclusively utilized in the initial KA model experiment.
5. To assess the effects of TPL on hippocampal neural circuits, the neural activities were recorded using a home‐made electrode (modified from our patent: ZL201822179088.4) in VGAT‐ChR2‐eYFP epileptic mice with or without 14‐day TPL treatments (50 μg/kg by intragastric administration). The home‐made electrode combined a 5 μL syringe, four recording wires, and a 200 μm optical fiber, which can be used for acute extracellular recording, drug delivery, and light delivery. Human recombinant IL‐1β (5 ng in 1 μL saline), interleukin‐1 receptor antagonist (IL‐1RA) (100 ng in 1 μL saline) or saline (1 μL) were injected using the 5 μL syringe at about 0.4 μL/min after a stable recording of baseline neural activities, as our previous study [12, 27]. To further investigate the role of the NF‐κB signaling pathway in the GABAergic “braking effect” TPCA‐1 (600 ng in 1 μL 1% DMSO) was also injected into the hippocampus before extracellular recording.

### 2.4 Seizure Models

#### 2.4.1 Maximal Electroshock Seizure Model

MES (0.2 s at 50 Hz, 15 mA) was delivered via ear clips using a rodent shocker (Hugo Sachs Elektronik, March‐Hugstetten, Germany) and produced a generalized seizure (GS) characterized by an initial tonic extension of the fore‐ and hindlimbs, followed by the clonic jerking of the musculature. The motor convulsion pattern was analyzed as a measure of the seizure severity, which was assigned scores based on the extent of the spread of tonic extension: 0, absence of forelimb extension; 1, complete forelimb extension without hindlimb extension; 2, complete forelimb extension with partial hindlimb extension; and 3, complete fore‐ and hindlimb extension (with hindlimbs fully extended parallel to the tail). The threshold for MES seizures was performed as in our previous study [28], the stimulus current intensity began at 5 mA (0.2 s at 50 Hz) and was varied by an “up‐and‐down” method. That is, the current was lowered 1 mA if the preceding shock caused tonic hindlimb extension or was raised 1 mA if not.

#### 2.4.2 PTZ‐Induced Seizure Model

PTZ was administered intraperitoneally at doses of 40 mg/kg for kindled mice and 60 mg/kg for normal mice, followed by 30‐min behavioral observation. Seizure severity was scored as follows: (0) no response; (1) ear and facial twitching; (2) myoclonic body jerks; (3) clonic forelimb convulsions; (4) generalized clonic convulsions and turning onto the side; (5) generalized clonic–tonic convulsions; and (6) death within 30 min. A trained observer, who was unaware of the experimental groupings, scored seizure severity. The dose threshold for PTZ was determined by an injection of 20 mg/kg followed by an injection of 10 mg/kg every 15 min until a generalized seizure occurred as in our previous studies [29].

#### 2.4.3 Corneal (6 Hz) Kindling Model

The kindling model was conducted according to the protocol described previously [30]. Mice were stimulated twice daily through corneal electrodes connected to an ECT Unit 57,800 stimulator (Ugo Basile) with a current intensity of 44 mA, 0.2 ms monopolar pulses at 6 Hz for a 3 s duration, which initially induces only focal seizures. Seizure severity was assessed after each stimulation according to Racine's scale. For testing the effects of TPL on corneal kindling, water or TPL was given once per day, 30 min prior to the first kindling stimulation.

#### 2.4.4 KA‐Induced Chronic Epilepsy Model

The KA‐induced chronic epilepsy model was established as in our previous study. Briefly, under anesthesia induced by sodium pentobarbital (60 mg/kg i.p.), KA (0.25 μg in 0.5 μL saline) was stereotaxically injected into the right dorsal hippocampus (AP: −2.0 mm; ML: +1.5 mm; DV: −2.0 mm). After injection, the needle of the microsyringe was maintained for an additional 5 min to limit reflux.

### 2.5 Surgery

Mice were anesthetized with sodium pentobarbital (60 mg/kg i.p.) and bipolar electrodes were then implanted into the dorsal hippocampus for EEG recording as described in previous studies [12, 27]. Briefly, a bipolar electrode was implanted in the dorsal hippocampus (AP: −2.0 mm; ML: +1.5 mm; DV: −2.0 mm) according to a mouse brain atlas [28], and a ground screw electrode was fixed above the cerebellum. The bipolar electrodes were made of twisted teflon‐coated tungsten wires (795,500; diameter, 0.05 mm; A.M. Systems). All these mice were treated with TPL for 14 days after recovery from surgery.

### 2.6 EEG Recording and Analysis

After 1 week of recovery, mice were monitored by video‐EEG for 8 h every day in an open field cage (40 cm × 40 cm × 40 cm). All mice still received treatment during recovery and EEG recording periods. Raw EEG signals in a unipolar setting were recorded with band‐pass filters spanning DC to 100 Hz and sampled at 1000 Hz with an online 50‐Hz line‐noise‐cancellation algorithm. Epileptiform discharges were defined as values 4.5 times higher than a threshold of the standard deviation of EEG signals in LabChart 8 software, as in a previous study [31]. Artificial events were manually removed.

### 2.7 Extracellular Recording and Optogenetics

Neuronal activity was sampled and analyzed as previously described [32, 33]. Briefly, the electrode was slowly lowered into the dorsal hippocampus (AP: −2.0 mm; ML: +1.5 mm; DV: −1.6 mm). The electrode was further lowered at 1 μm/s from 1.6 mm to 2.4 mm until single‐unit neuronal activities were recorded. Neuronal activity was sampled using a Cerebus acquisition system (Blackrock Microsystems) grounded and referenced to screws above the cerebellum with an online 50‐Hz line‐noise‐cancellation algorithm. Recorded neuronal data were re‐sorted offline using offline sorter software (Plexon Inc) and analyzed using Neuroexplorer 5.0 software (NEX Technologies Int Inc). Putative principal neurons (PNs) were distinguished based on their response to the optogenetic stimulation, firing rate, and waveform as our previous studies [31, 32, 33].

Laser light (optogenetical stimulation) was delivered through a 200‐μm diameter optic fiber connected to lasers (IKECOOL Laser, 473 nm) by fiber‐optic joints (Doric Lenses). The fiber was cut flat, and the laser power was adjusted to ~10.0 mW. We used a 5 s train of light stimulation at 473 nm, 20 Hz, and 5 ms per pulse to assist in identifying the types of neurons in VGAT‐ChR2‐eYFP mice. We used a 250 s train of light stimulation at 473 nm, 0.2 Hz, and 5 ms per pulse to evaluate the GABAergic “braking effect” of the hippocampus in VGAT‐ChR2‐eYFP mice.

### 2.8 Novel Object Recognition Test and Novel Location Recognition Test

After EEG recording (about 28‐day treatments), the novel object recognition test (NORT) and novel location recognition test (NLRT) were performed as in our previous studies [34]. Briefly, each mouse was exposed to two cuboid objects that were identical for 10 min to train them. The two objects were placed in the rear corners of the square arena. After a 30‐min retention interval in the home cage, the NORT or NLRT was conducted. Exploration was defined as touching the object with the nose or pointing its nose toward the object at a distance of < 2 cm; actions like climbing, sitting on, or turning around an object were not classified as exploration. The recognition index (%) of object placement was calculated as [exploration time of the novel object or place − exploration time of the familiar object or place]/[exploration time in both objects or places] × 100. The novel objects used were cylindrical objects. Olfactory cues were removed from objects and arenas by cleaning them with 75% ethanol before the next test.

### 2.9 Sample Preparation and Cytokine Measurement

At the conclusion of NORT and NLRT, the mice were anesthetized using sodium pentobarbital (60 mg/kg, i.p.). Subsequently, the brain was rapidly dissected following the intracardiac perfusion with saline. The hippocampus was rapidly separated out from the brain, then immediately frozen in liquid nitrogen and stored at −80°C. Total protein was extracted from the hippocampus. Briefly, frozen hippocampus was homogenized in ice‐cold extraction buffer containing protease inhibitor mixture (Sigma‐Aldrich). Then, samples were maintained at 4°C for 2 h, followed by centrifugation at 12,000 × g for 20 min. Supernatants were collected and analyzed for cytokine measurement. IL‐1β was tested using an enzyme‐linked immunosorbent assay (ELISA) kit (Multi Sciences, China, # EK201B/3–96) according to the manufacturer's instructions.

### 2.10 Immunofluorescence Staining

Mice were euthanized with an overdose of 1% sodium pentobarbital and perfused with icy saline and 4% paraformaldehyde (PFA). The brains were fixed for 48 h in 4% PFA, dehydrated for 24 h in 20% and 24 h in 30% sucrose solution. The brains were sliced into 16 μm sections. The sections were incubated with anti‐GFAP antibody (1:400, GeneTex; #GTX108711) at 4°C overnight. Then, the sections were thoroughly washed and incubated at 37°C for 30 min with an Alexa Fluor 647‐conjugated antibody (1:200, Abcam; #ab150083). After washing, the cell nuclei were counterstained with DAPI (Meilunbio; #MA0222‐L). The sections were observed using an Olympus VS120–S6–W microscope or a Zeiss LSM880 laser scanning confocal microscope. The volume of GFAP^+^ astrocytes and VGAT^+^ neurons was quantified by Imaris software (v.10.0, Bitplane). High‐resolution confocal images were processed using background filtering GFAP and VGAT. Surfaces were smoothed by 0.15 μm. The volume of GFAP and VGAT remodeling surface was recorded.

### 2.11 Western Blotting

The hippocampus tissue prepared from mice was generated by homogenization and sonication in RIPA buffer. The protein concentration was determined by BCA assay. Equal amounts of proteins (40 ng) were separated by SDS‐PAGE and transferred to a PVDF membrane. After blocking with 5% skim milk at room temperature for 1 h, the membranes were incubated with primary antibodies (GFAP (1:1000, Abcam); GAD 65/67 (1:1000; Abcam); β‐actin (1:4000, Cell signaling); and GAPDH (1:10000, Huabio, China)) at 4°C overnight. After washing with Tris‐buffered saline/1% Tween‐20 (TBS‐T) for 0.5 h, the membranes were incubated with the appropriate secondary antibody (1:5000, LI‐COR) for 2 h. After washing with TBS‐T buffer again, the film was scanned in the Odyssey fluorescence imaging system, and quantitative analysis was performed by the Image J software.

### 2.12 Target of TPL and Data Acquisition of Genes Related to Epilepsy

The targets of TPL were searched from the TCMID database (http://www.megabionet.org/tcmid/), and the medium confidence targets whose STITCH score was more than 0.4 were selected for further analysis. Besides, the epilepsy‐related genes were collected from previous studies, which were generated (GSE63808) from the hippocampus of 129 patients with temporal lobe epilepsy (TLE). The protein–protein interaction (PPI) network was generated by the String plugin in Cytoscape 3.2.1, and the clusters of PPI networks were further analyzed by the Molecular Complex Detection (MCODE) plugin.

### 2.13 RNA‐Sequencing

RNA was isolated from the bilateral dorsal hippocampus using TRIzol Reagent. RNA quality was assessed by 2100 Bioanalyser. Only high‐quality RNA samples (OD 260/280 = 1.8~2.2, OD 260/230 ≥ 2.0, RIN ≥ 8.0, 28S:18S ≥ 1.0, > 1 μg) were used to construct the sequencing library. The transcriptome library was prepared following the TruSeq TM RNA sample preparation Kit from Illumina (San Diego, CA) using 1 μg of total RNA. Paired‐end RNA‐seq sequencing libraries were sequenced with the Illumina NovaSeq 6000 sequencer (2 × 150 bp read length). The raw paired‐end reads were trimmed and quality controlled by fastp (https://github.com/OpenGene/fastp) with default parameters. Then clean reads were separately aligned to the reference genome with orientation mode using HISAT2 (http://ccb.jhu.edu/software/hisat2/index.shtml) software. The mapped reads of each sample were assembled by StringTie (https://ccb.jhu.edu/software/stringtie/) in a reference‐based approach. DEG analysis was performed using the DESeq2. The *ggplot2* and *pheatmap* packages were used for the visualization and generation of volcano plots and heatmaps, respectively, to facilitate an intuitive understanding of the data. We conducted the Kyoto Encyclopedia of Genes and Genomes (KEGG) pathway analysis with the *clusterProfiler* package of R. *p* < 0.05 was taken to indicate statistical significance.

### 2.14 Statistical Analysis

The data in this study are presented as mean ± SEM. Statistical comparisons were performed using GraphPad Prism 8.0 software. The normality of data distribution was assessed using the Shapiro–Wilk test, while homogeneity of variances was verified via *F*‐test or Brown‐Forsythe test when normality assumptions were met. For two‐group comparisons, Student's *t*‐test or Mann–Whitney test was used. Multi‐group comparisons (three or four groups) were analyzed using one‐way ANOVA (with Tukey test for post hoc comparison) or Kruskal‐Wallis test (with Dunn's test for post hoc comparison). Seizure stage development in the corneal kindling model was analyzed using two‐way ANOVA (with Least Significant Difference post hoc test). The percentage of non‐kindled mice was assessed using the log‐rank (Mantel‐Cox) test, and the incidence of generalized seizures was analyzed by Fisher's exact test. *p* < 0.05 was considered statistically significant. Detailed statistical descriptions for all figures are provided in Table S1.

## 3 Results

### 3.1 TPL Had no Effect on Acute Seizures in Normal Mice

**FIGURE 1:**
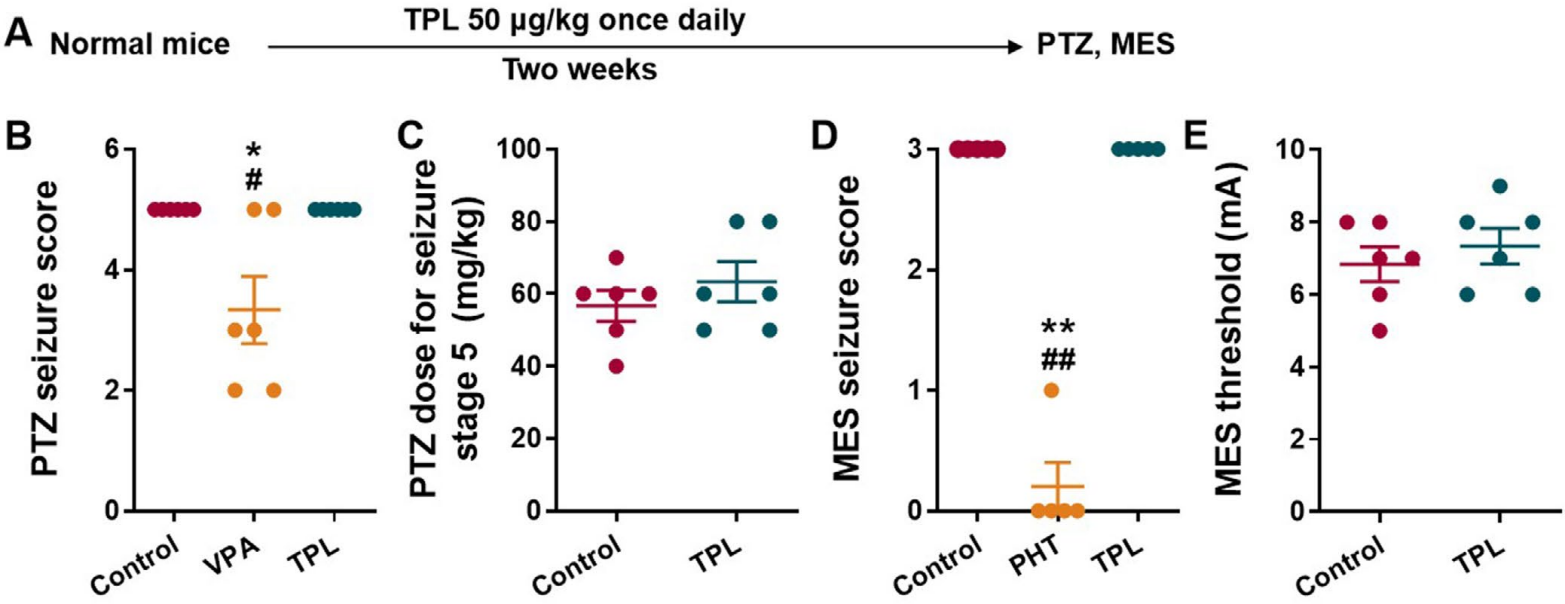
TPL had no effect on acute seizures in normal mice. (A) Schematic diagram of experimental design to prove the effect of TPL in PTZ model and MES model; (B) Effect of TPL on seizure scores in PTZ (60 mg/kg) model (*n* = 6 mice per group); (C) Effect of TPL on seizure thresholds in PTZ model (*n* = 6 mice per group); (D) Effect of TPL on seizure scores in MES (15 mA) model (*n* = 5 mice per group); (E) Effect of TPL on seizure threshold in MES model (*n* = 6 mice per group). Data are presented as mean ± SEM. Kruskal‐Wallis test was used for B and D, Student's t‐test was used for C and E. *p < 0.05, **p < 0.01 compared with Control. ^#^ *p* < 0.05, ^##^ *p* < 0.01 compared with TPL.

To assess the antiepileptic effect of TPL, we used two classic acute seizure models, the PTZ‐induced seizure model and the MES model. We found TPL, 50 μg/kg daily for 2 weeks, had no effect on the seizure scores or seizure thresholds in PTZ and MES models in normal mice (*p* > 0.05 for all, Figure 1A–E), while classic antiseizure medications (ASMs), valproic acid sodium (VPA) and phenytoin sodium (PHT) reduced the seizure scores in PTZ and MES models in normal mice (*p* < 0.05 for Figure 1B, *p* < 0.01 for Figure 1D), respectively.

### 3.2 TPL Retarded Corneal Kindling Acquisition in Mice

**FIGURE 2:**
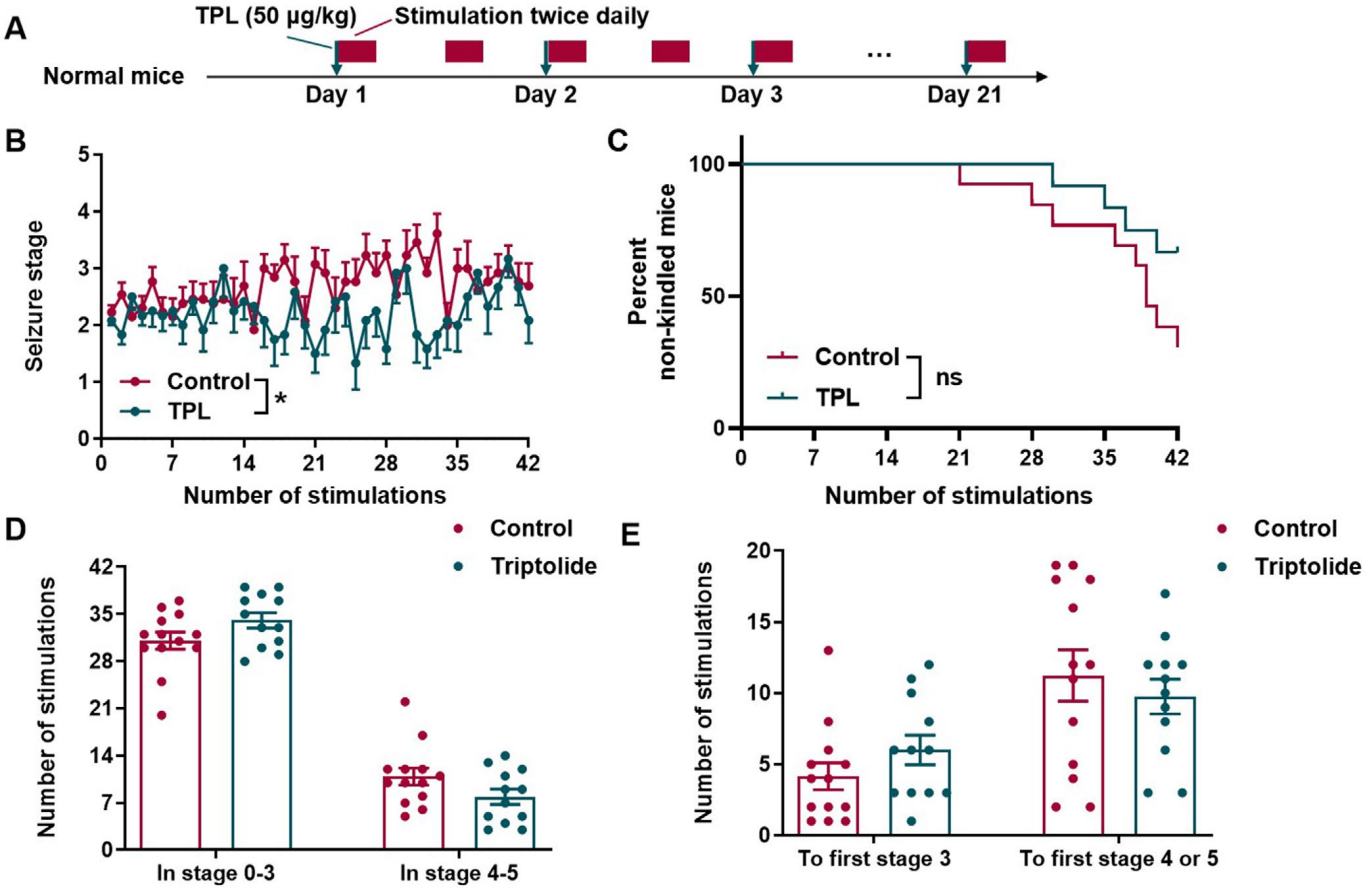
TPL retarded corneal kindling acquisition in mice. (A) Schematic diagram of experimental design to prove the effect of TPL on corneal kindling acquisition; (B) The development of seizure stage during 6‐Hz corneal kindling acquisition (Control, *n* = 13 mice; TPL, *n* = 12 mice); (C) The percent of non‐kindled mice (Control, *n* = 13 mice; TPL, *n* = 12 mice); (D) Number of stimulations stayed in 0 to 3seizure stage or GS during kindling acquisition (Control, *n* = 13 mice; TPL, *n* = 12 mice); (E) Number of stimulations needed to reach stage 3 or GS during kindling acquisition (Control, *n* = 13 mice; TPL, *n* = 12 mice). Data are presented as mean ± SEM. Two‐way ANOVA (with LSD test for post hoc comparison) was used for B, Log‐rank (Mantel‐Cox) test was used for C, Student's t‐test and Mann Whitney test were used for D and E. *p < 0.05 compared with Control.

To assess the antiepileptic effect of TPL in the chronic seizure, we used a chronic epilepsy model, the corneal (6 Hz) kindling model. We conducted 6‐Hz corneal stimulations twice daily for 21 days on two groups of mice; the mice were given TPL 50 μg/kg or water 0.1 mL/10 g before the first stimulation daily (Figure 2A). We found TPL, 50 μg/kg once daily, retarded the kindling acquisition (*p* < 0.05, Figure 2B) and had a slightly higher percent of non‐kindled mice (*p* = 0.0954, Figure 2C) the in corneal (6 Hz) kindling model. Moreover, TPL‐treated mice exhibited a slightly higher frequency of remaining in the 0–3 seizure stage (*p* = 0.0928, Figure 2D) and needed a little bit more stimulations to reach the first stage 3 (*p* = 0.1532, Figure 2E) compared to the mice received water. Of note, the kindled seizures in corneal (6 Hz) kindling model were resistant to PHT and VPA [35].

### 3.3 TPL Reduced the Severity of Seizure in Corneal Kindled Mice

**FIGURE 3:**
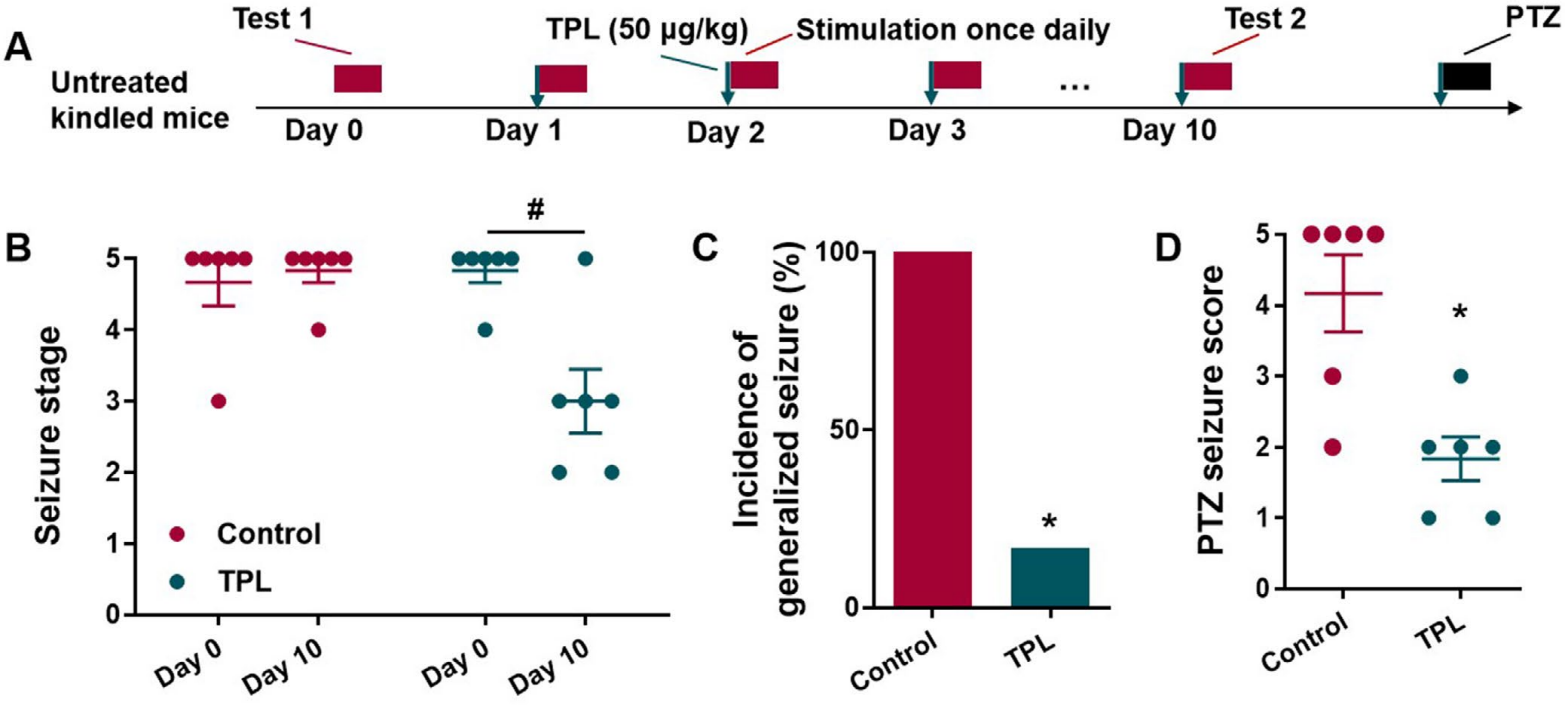
TPL reduced the severity of seizure in corneal kindled mice. (A) Schematic diagram of experimental design to prove the effect of TPL on PTZ (40 mg/kg) model in corneal kindled mice; (B) Effect of TPL on seizure stage (*n* = 6 mice per group); (C) Effect of TPL on incidence of generalized seizure (*n* = 6 mice per group); (D) Effect of TPL on seizure score (*n* = 6 mice per group). Data are presented as mean ± SEM. Mann Whitney test was used for B and D, Fisher's exact test was used for C. ^#^ *p* < 0.05 compared with Day 0. *p < 0.05 compared with Control.

To investigate whether TPL has an impact on epilepsy susceptibility in corneal kindled mice, the kindled mice were given TPL 50 μg/kg or water 0.1 mL/10 g before stimulation daily; the seizure stage was recorded on day 0 and day 10 (Figure 3A). TPL reduced the seizure stage in corneal kindled mice (*p* < 0.05, Figure 3B). Meanwhile, TPL inhibited the incidence of GS (*p* < 0.05, Figure 3C) and seizure score (*p* < 0.05, Figure 3D) of PTZ‐induced seizure in the corneal kindled mice.

### 3.4 TPL Acted Disease Modifying Effect in KA Mouse Model of Epilepsy

**FIGURE 4:**
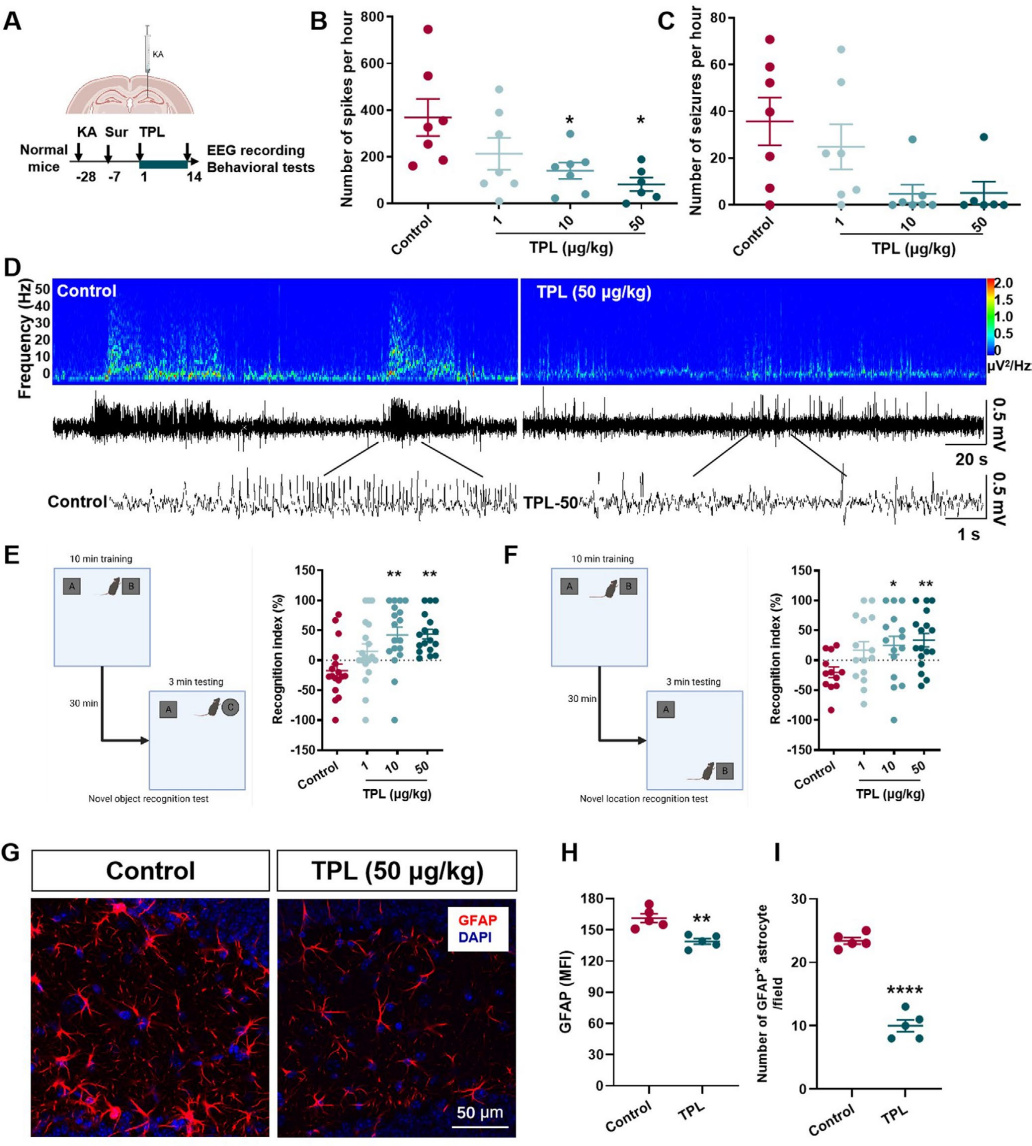
TPL acted disease modifying effect in KA mouse model of epilepsy. (A) Schematic diagram of experimental design to prove the effect of TPL on KA model; (B) The effect of TPL on interictal discharges in established KA model (Control, *n* = 7 mice; TPL (1 μg/kg), *n* = 7 mice; TPL (10 μg/kg), *n* = 7 mice; TPL (50 μg/kg), *n* = 6 mice); (C) The effect of TPL on epileptic seizures in established KA model (Control, *n* = 7 mice; TPL (1 μg/kg), *n* = 7 mice; TPL (10 μg/kg), *n* = 7 mice; TPL (50 μg/kg), *n* = 6 mice); (D) Representative EEGs of KA mice and TPL‐treated KA mice; (E) Recongnition index in novel object recognition test (Control, *n* = 17 mice; TPL (1 μg/kg), *n* = 19 mice; TPL (10 μg/kg), *n* = 18 mice; TPL (50 μg/kg), *n* = 17 mice); (F) Recongnition index in novel location recognition test (Control, *n* = 12 mice; TPL (1 μg/kg), *n* = 15 mice; TPL (10 μg/kg), *n* = 15 mice; TPL (50 μg/kg), *n* = 17 mice); (G) Representative immunofluorescence staining of GFAP (red) in hippocampus, scale bar = 50 μm; (H) Mean fluorescence intensity (MFI) of the GFAP in DG region (*n* = 5 mice per group); (I) The number of GFAP^+^ astrocytes per field (*n* = 5 mice per group). Data are presented as mean ± SEM. One‐way ANOVA (with Tukey test for post hoc comparison) was used for B and F, Kruskal‐Wallis test was used for C and E, Student's t‐test was used for H‐I. *p < 0.05, **p < 0.01, and ****p < 0.0001 compared with Control.

To further evaluate the antiepileptic effect of TPL in epileptic conditions, we first used an established KA model that experiences spontaneous recurrent seizures (Figure 4A). We found TPL dose‐dependently reduced the frequency of epileptic seizure events in the established KA model. TPL at 1 μg/kg only slightly reduced the frequency of burst events, while TPL at 10 or 50 μg/kg reduced the frequency of inter‐ictal spikes (*p* < 0.05, Figure 4B) and epileptic seizures (*p* = 0.1276 for Control vs. TPL 10 μg/kg, *p* = 0.1401 for Control vs. TPL 50 μg/kg, Figure 4C). Representative EEGs were shown in Figure 4D.

Cognitive impairment has been considered an integral manifestation of epileptic models and patients with TLE [36, 37]. We used the novel object recognition test and location recognition test to evaluate whether TPL had a beneficial effect on the cognitive impairment in the established KA model. TPL dose‐dependently improved the recognition memory impairment in the novel object recognition test (*p* < 0.01 for Control vs. TPL 10 μg/kg, *p* < 0.01 for Control vs. TPL 50 μg/kg, Figure 4E) and location recognition test (*p* < 0.05 for Control vs. TPL 10 μg/kg, *p* < 0.01 for Control vs. TPL 50 μg/kg, Figure 4F).

As astrocytosis is a hallmark of TLE, we tested the effect of TPL on the astrocytosis in the epileptic hippocampus in the KA model (Figure 4G). TPL significantly inhibited the astrocytosis in the DG region (*p* < 0.01 for Figure 4H, *p* < 0.0001 for Figure 4I).

### 3.5 TPL Enhanced GABAergic “Braking Effect” in KA Mouse Model of Epilepsy

**FIGURE 5:**
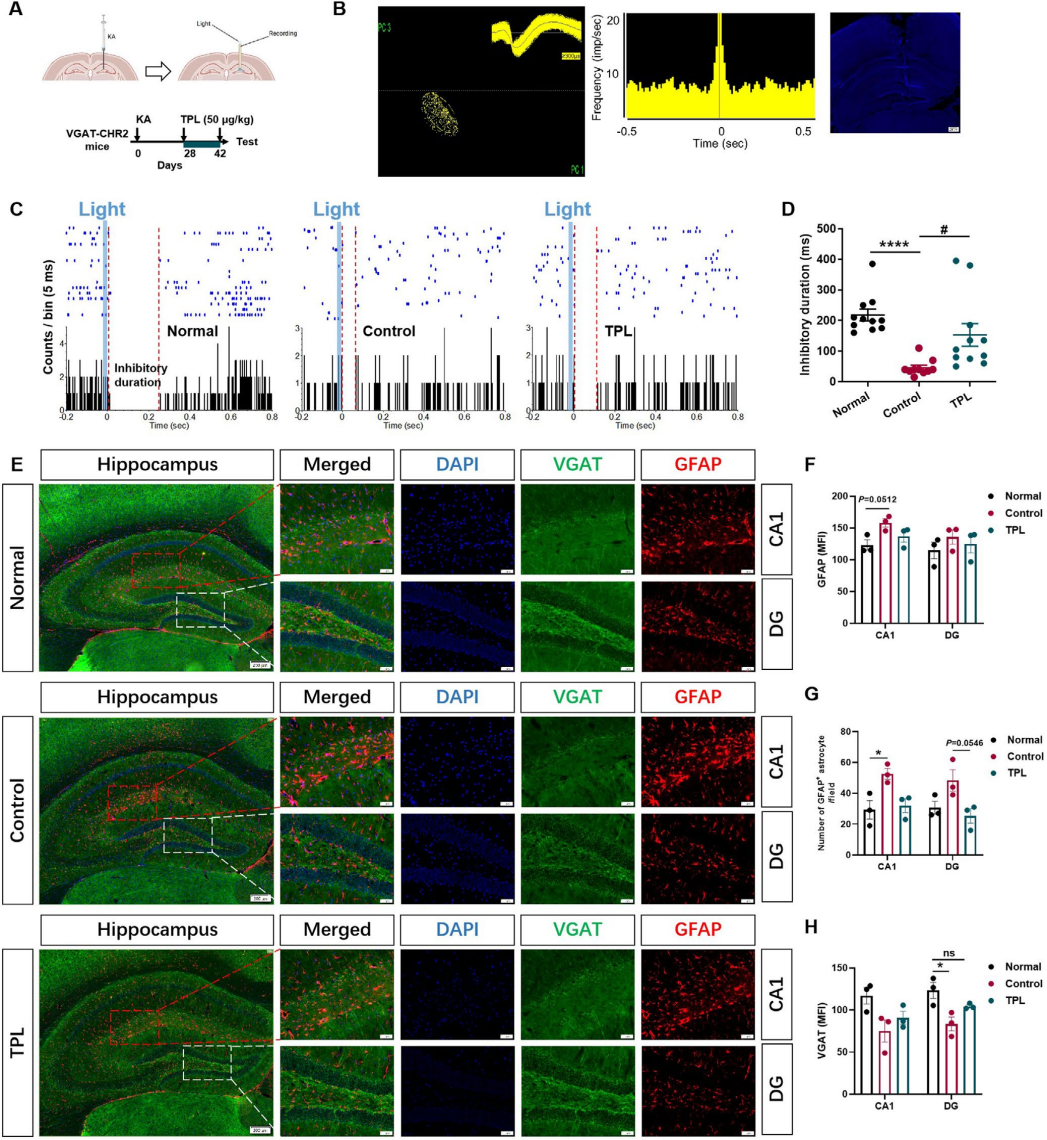
TPL enhanced GABAergic “braking effect” in KA mouse model of epilepsy. (A) Schematic diagram of optogenetical stimulation and microelectrode recording on KA model; (B) Clusters view and waveform of recorded neurons (left); autocorrelation of recorded neurons (middle); histological image showing the placement of electrode and optic fiber in dorsal hippocampal DG for extracellular recording (right, Blue‐DAPI); (C) Peri‐event raster histograms of the event‐related response of neurons to optogenetical stimulation; (D) The duration of the light‐induced “braking effect” (Normal, *n* = 11 cells; Control, *n* = 10 cells; TPL, *n* = 11 cells). (E) Example images showing co‐localization of VGAT‐EYFP with astrocytes (GFAP) in hippocampus (scale bar = 200 μm), CA1 and DG region (scale bar = 50 μm); (F) The mean fluorescence intensity of GFAP in CA1 and DG region (Normal, *n* = 3 mice; Control, *n* = 3 mice; TPL, *n* = 3 mice); (G) The number of GFAP^+^ astrocytes CA1 and DG region (Normal, *n* = 3 mice; Control, *n* = 3 mice; TPL, *n* = 3 mice); (H) The mean fluorescence intensity of VGAT in CA1 and DG region (Normal, *n* = 3 mice; Control, *n* = 3 mice; TPL, *n* = 3 mice). Normal means sham operated VGAT‐CHR2‐EYFP mice administrated with solvent at 0.1 mL/10 g; Control means VGAT‐CHR2‐EYFP epileptic mice administrated with solvent at 0.1 mL/10 g; TPL means VGAT‐CHR2‐EYFP epileptic mice administrated with TPL at 50 μg/kg. Data are presented as mean ± SEM. Kruskal‐Wallis test was used for D. *p < 0.05, ****p < 0.0001 compared with Normal. ^#^ *p* < 0.05 compared with Control.

The degeneration of hippocampal GABAergic interneurons is a characteristic pathology in patients with medial temporal lobe epilepsy (mTLE) and the KA mouse model of TLE [31]. To investigate the effect of TPL on hippocampal GABAergic interneurons, we used optogenetics combined with microelectrode recording in the VGAT‐CHR2‐EYFP mice (Figure 5A). The firing frequency of glutamatergic neurons is generally less than 10 Hz, with a maximum half‐wave width of ≥ 0.30 ms and a sharp autocorrelation analysis. Based on the recorded characteristics of the neural discharge waveform and autocorrelation analysis, it can be determined whether it is a glutamatergic neuron. The histological image with DAPI showing the location of extracellular recording (Figure 5B). The example neurons responded to illumination are shown in Figure 5C. It was found that the selective activation of VGAT‐positive GABAergic nerve cells the in DG region by light stimulation can induce a “braking effect” on hippocampal glutamatergic neurons in normal animals, but this effect is significantly weakened in epileptic mice. TPL intervention enhanced the “braking effect” the in DG region in epileptic mice (*p* < 0.0001 for Normal vs. Control, *p* < 0.05 for Control vs. TPL, Figure 5D). The changes in the morphology and number of astrocytes and GABAergic neurons may cause the difference the “braking effect”. Immunofluorescence staining the of CA1 and DG regions were shown in Figure 5E. Using the VGAT‐CHR2‐EYFP epileptic mice, it was found that the KA modeling increased the mean fluorescence intensity (*p* = 0.0512 for Normal vs. Control in CA1, Figure 5F) and the number of GFAP^+^ astrocytes (*p* < 0.05 for Normal vs. Control in CA1, *p* = 0.0546 for Normal vs. Control in DG, Figure 5G), and decreased the mean fluorescence intensity of VGAT^+^ GABA neurons in mice, and this effect could be significantly reversed by TPL (*p* < 0.05 for Normal vs. Control in DG, Figure 5H).

### 3.6 TPL Reverses Astrocytic and GABAeric Neuronal Dysregulation in KA Model

**FIGURE 6:**
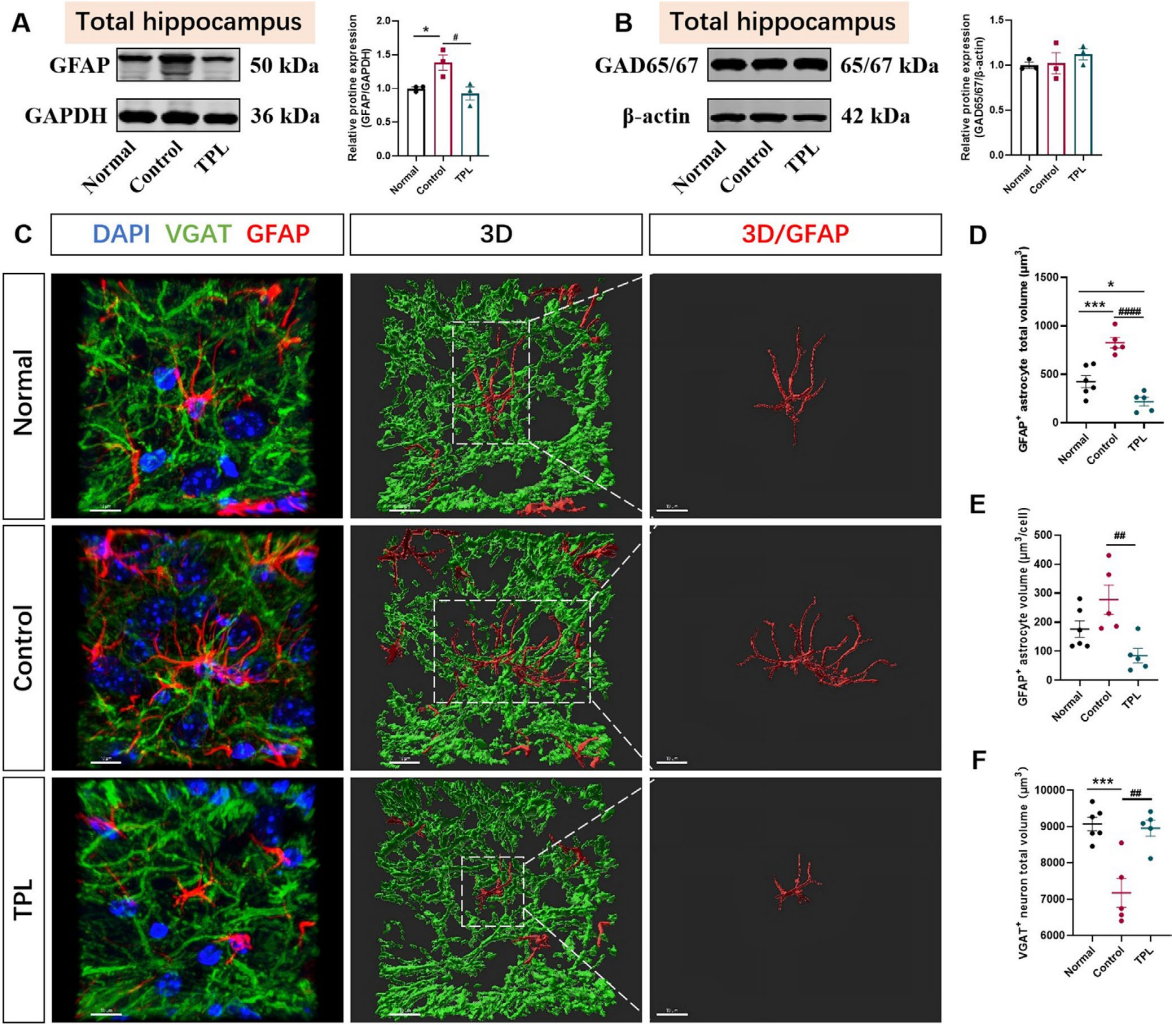
TPL reverses astrocytic and GABAergic neuronal dysregulation in the KA model. (A) Representative WB results of GFAP expression and related quantitative statistical plots. (B) Representative WB results of GAD65/67 expression and related quantitative statistical plots. (C) 3D reconstructions of GFAP^+^ astrocytes and VGAT^+^ GABAergic neurons (scale bar = 10 μm); (D) The total volume of GFAP^+^ astrocytes (Normal, *n* = 6 fields; Control, *n* = 5 fields; TPL, *n* = 5 fields). (E) The volume of single GFAP^+^ astrocytes (Normal, *n* = 6 cells; Control, *n* = 5 cells; TPL, *n* = 5 cells). (F) The total volume of VGAT^+^ GABAergic neurons (Normal, *n* = 6 fields; Control, *n* = 6 fields; TPL, *n* = 6 fields). Normal means sham‐operated VGAT‐CHR2‐EYFP mice administrated with solvent at 0.1 mL/10 g; Control means VGAT‐CHR2‐EYFP epileptic mice administrated with solvent at 0.1 mL/10 g; TPL means VGAT‐CHR2‐EYFP epileptic mice administrated with TPL at 50 μg/kg. Data are presented as mean ± SEM. One‐way ANOVA (with Tukey test for post hoc comparison) was used for A‐B and D‐F. *p < 0.05, ***p < 0.001 compared with Normal. ^#^ *p* < 0.05, ^##^ *p* < 0.01, ^####^ *p* < 0.0001 compared with Control.

To further elucidate the effects of astrocytes and GABAergic neurons in the KA model, we performed a Western blot on hippocampal tissue and a 3D reconstruction of immunofluorescence of the DG region. The Western blot results confirmed the effect of TPL on astrocytes (*p* < 0.05 for Normal vs. Control; *p* < 0.05 for Control vs. TPL, Figure 6A), but no significant regulation was observed on GDA65/67 protein levels (*p* > 0.05, Figure 6B). Furthermore, the results showed the total volume of GFAP^+^ astrocytes was increased after KA modeling, while TPL attenuated this increase (*p* < 0.001 for Normal vs. Control; *p* < 0.0001 for Control vs. TPL Figure 6D). We also analyzed the volumetric measurements of individual GFAP^+^ astrocytes and observed a consistent trend (*p* < 0.01 for Control vs. TPL, Figure 6E). The total volume of VGAT^+^ GABA neurons in the model group was decreased after KA modeling (*p* < 0.0001 for Normal vs. Control; *p* < 0.01 for Control vs. TPL, Figure 6F), which may have contributed to the “braking effect”. This discrepancy with the immunofluorescence results may be attributed to the fact that the Western blot detected protein levels across the whole hippocampus rather than the local lesioned area.

### 3.7 IL‐1β and Related Inflammatory Signaling Impaired Hippocampal GABAergic “Braking Effect” in Normal Mice

**FIGURE 7:**
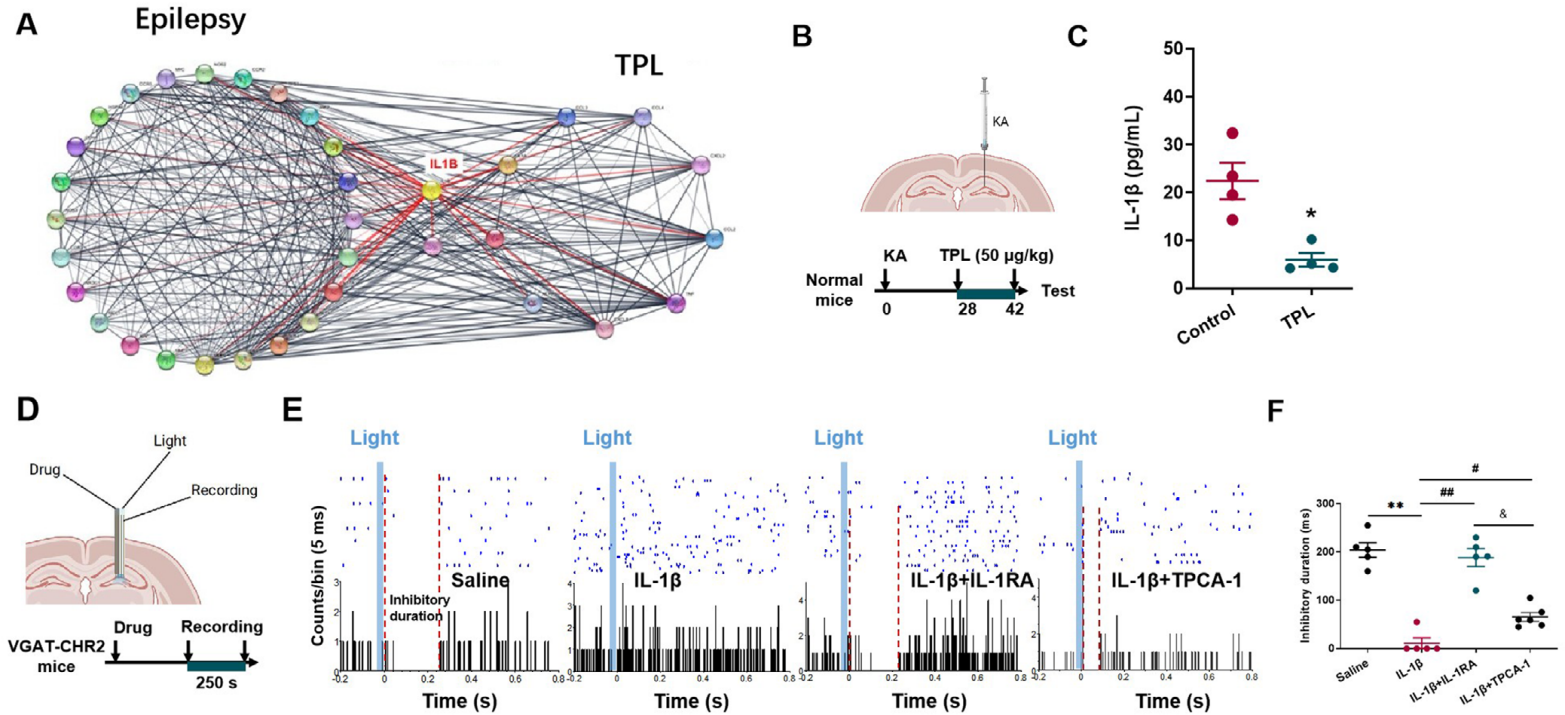
IL‐1β impaired hippocampal GABAergic “braking effect” in normal mice. (A) PPI network for the core clusters of targets of TPL and the genes co‐expression network from hippocampal tissue of TLE patients; (B) Schematic diagram of experimental design to prove the effect of TPL in KA model; (C) The level of IL‐1β in hippocampus (Control, *n* = 4 mice; TPL, *n* = 4 mice); (D) Schematic diagram of optogenetical stimulation, microelectrode recording, and intrahippocampal injection. (E) Peri‐event raster histograms of the event‐related response of neurons to optogenetical stimulation; (F) The inhibitory duration of light‐induced “braking effect” (Saline, *n* = 5 cells; IL‐1β, *n* = 5 cells; IL‐1β + IL‐1RA, *n* = 5 cells, IL‐1β + TPCA‐1, *n* = 6 cells). Data are presented as mean ± SEM. Mann‐Whitney test was used for C. Kruskal‐Wallis test was used for F. *p < 0.05 compared with Control in C. **p < 0.01 compared with Saline, ^#^ *p* < 0.05, ^##^ *p* < 0.01 compared with IL‐1β, ^&^ *p* < 0.05 compared with IL‐1β + IL‐1RA in F.

To predict the potential key targets of TPL for the treatment of epilepsy, we collected the targets of TPL (data from TCMID database) and the genes co‐expression network from hippocampal tissue of TLE patients (data from Johnson MR et al. [38]). As shown in the PPI network of the two core clusters, IL‐1β may be the key target for the antiepileptic effect of TPL (Figure 7A). Subsequently, we validated the effect of TPL on IL‐1β (Figure 7B), and TPL indeed reduced the level of IL‐1β in the hippocampus (*p* < 0.05, Figure 7C).

To confirm the role of IL‐1β in this hippocampal GABAergic “braking effect”, we injected the human recombinant IL‐1β (5 ng in 1 μL saline) into the DG region of normal mice using a homemade electrode, as shown in Figure 7D. IL‐1β directly weakened the above “braking effect”, while the IL‐1RA (100 ng in 1 μL saline) could partially weaken the effect of IL‐1β. Notably, the NF‐κB inhibitor, TPCA‐1, also partially weakened the effect of IL‐1β (*p* < 0.01 for Saline vs. IL‐1β & IL‐1β vs. IL‐1β + IL‐1RA. *p* < 0.05 for IL‐1β vs. IL‐1β + TPCA‐1 & IL‐1β + IL‐1RA vs. IL‐1β + TPCA‐1, Figure 7E,F).

### 3.8 TPL Inhibited Inflammation‐Related Signal Pathways for Epilepsy Protection

**FIGURE 8:**
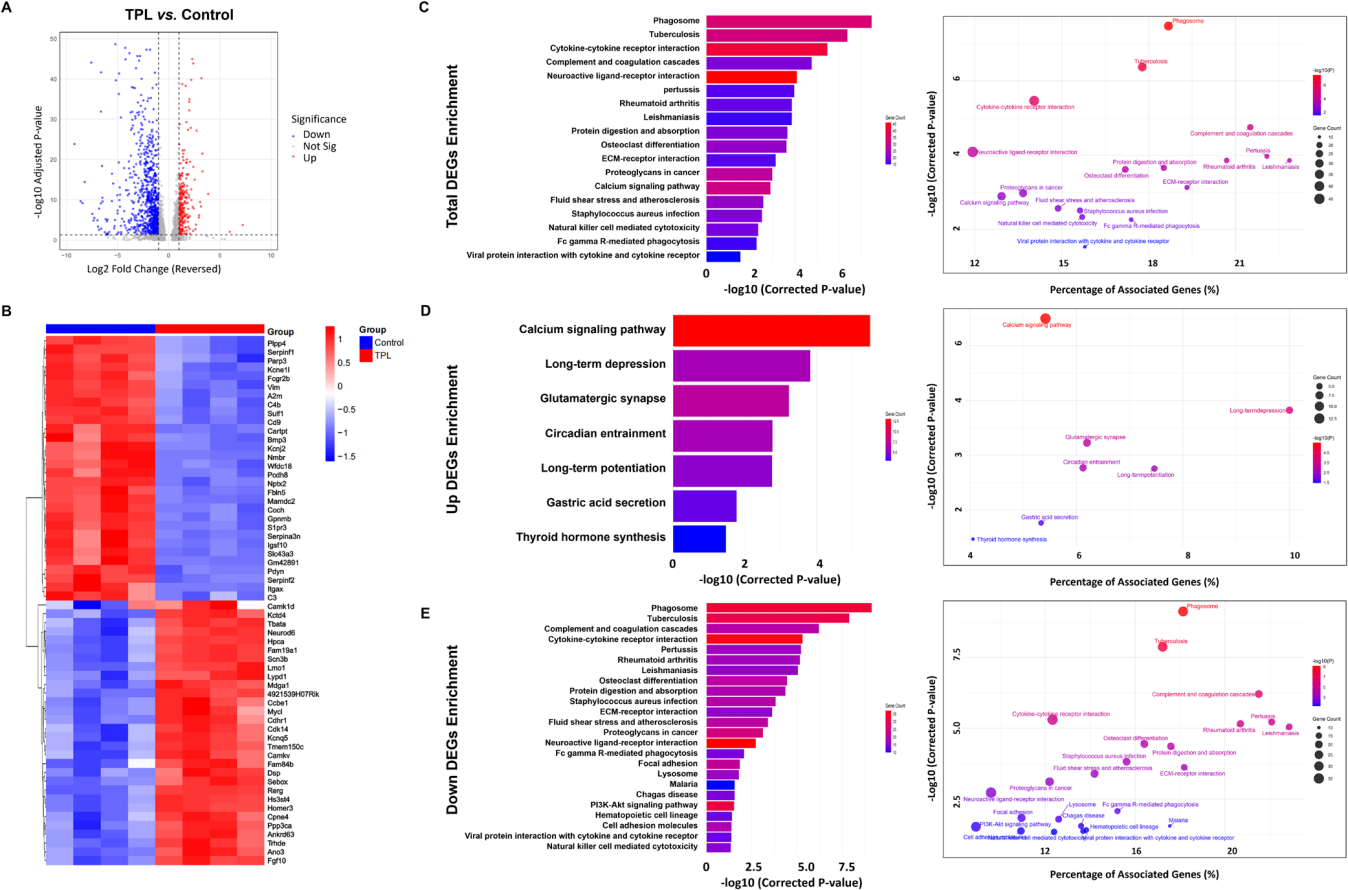
TPL inhibited inflammation‐related signal pathways for epilepsy protection. (A) Volcano plot of all genes from the comparison of TPL to control (Control, *n* = 4 mice; TPL, *n* = 4 mice); (B) The heatmap of differential genes significantly changed after TPL adminstration (Control, *n* = 4 mice; TPL, *n* = 4 mice); (C) KEGG pathway enrichment analysis of total DEGs (left: Bar plot, right: Bubble plot); (D) KEGG pathway enrichment analysis of up‐regulated DEGs (left: Bar plot, right: Bubble plot); (E) KEGG pathway enrichment analysis of down‐regulated DEGs (left: Bar plot, right: Bubble plot). *p* < 0.05 was taken to indicate statistical significance.

To gain further insight into the mechanism by which TPL modulates epilepsy in KA, total RNA was extracted from hippocampus tissues, and RNA sequencing was performed from control and TPL groups. Compared to the control group, differential gene expression analysis (≥ 1.0‐fold change, *q*‐values ≤ 0.05) revealed 1094 DEGs in the TPL group (Figure 8A and Table S2). The heat map analysis depicted the 30 most significant up‐ or down‐regulated DEGs between the control and TPL groups (Figure 8B). It revealed that TPL treatment significantly downregulated key neuroinflammatory markers (C3, C4b, Itgax) and neuronal activity regulators (Hpca, Homer3, Camkv), demonstrating its dual action on both suppressing neuroinflammation and protecting neural function. KEGG analyses showed that TPL effectively downregulated neuroinflammatory pathways including complement and coagulation cascades, and cytokine‐cytokine receptor interaction. Furthermore, TPL normalized the neuroactive ligand‐receptor interactions and calcium signaling pathway, suggesting its protective role in maintaining neuronal homeostasis and reducing hyperexcitability. These coordinated modulatory effects highlight the dual therapeutic potential of TPL in simultaneously suppressing neuroinflammation and protecting neural function in epilepsy (Figure 8C). Separate KEGG analysis of up‐ and down‐regulated DEGs revealed that up‐regulated DEGs were primarily enriched in pathways associated with neural signaling and synaptic plasticity (Figure 8D), whereas down‐regulated DEGs were primarily enriched in pathways associated with neuroinflammation (Figure 8E).

## 4 Discussion

A large body of evidence that has accumulated over the past decades strongly supports the role of inflammation in the pathophysiology of epilepsy. However, single‐target drugs acting on one of these inflammation pathways usually show limited antiepileptic effects. Here we evaluated the antiepileptic effect of TPL (a multiple‐target anti‐inflammation compound from TWHF) and explored its underlying mechanism at the neural circuit level in vivo. We used a corneal (6 Hz) kindling model, which is considered a model of drug‐resistant epilepsy. We were surprised to find that TPL retarded corneal kindling acquisition and reduced the severity of seizures in corneal kindled mice. Meanwhile, we used a KA mouse model of TLE, which is widely considered to closely mimic the human TLE condition in that the animals manifest chronic epilepsy with both recurrent spontaneous seizures and cognitive comorbidities. We mainly found that: (1) TPL reduced the number of seizures and improved cognitive performance in the KA mouse model of TLE; (2) TPL enhanced the GABAergic “braking effect” in epileptic mice; (3) Bioinformatics analysis predicts IL‐1β as a key target for the antiepileptic effect of TPL; (4) IL‐1β impaired hippocampal GABAergic “braking effect” in normal mice, which was almost totally restored by pretreatment with IL‐1RA and partly restored by pretreatment with TPCA‐1 (an IKK‐2/NF‐κB inhibitor). Thus, our results indicate TPL may be a candidate compound for epileptic treatment via targeting the neuroinflammation‐related hippocampal neural circuit dysfunction in mice.

The antiepileptic effects of TPL also have been proposed by several studies [39, 40] and reviewed by Cui et al. 2022 [41]. Specifically, Pan et al. employed an acute KA (intraperitoneal) rat model to examine the modulation of TPL on voltage‐gated potassium channel Kv1.1 expression, while Sun et al. focused on the regulation of TPL on MHC II and CIITA expression in KA‐stimulated BV‐2 microglial cells, without extending to in vivo validation. In our study, we confirmed the antiepileptic effects of TPL in both corneal (6 Hz) kindling model and chronic KA mice model, while our findings showed that TPL did not exhibit significant effects on acute PTZ and MES models. The PTZ and MES models are commonly used acute seizure models, often employed in the preliminary screening of antiepileptic drugs due to their simplicity and ease of operation. However, in this study, TPL showed no significant efficacy in these acute models. The corneal (6 Hz) kindling model is a tested tool for discovering ASMs for drug‐resistant epilepsy [42]. We demonstrated that TPL not only retarded the kindling development but also suppressed kindled seizures in kindled mice, suggesting that its antiepileptic effects depend on the presence of progressive and chronic epilepsy. The chronic KA model is a commonly used TLE model, and its pathological characteristics closely resemble those of clinical TLE patients, including hippocampal neuronal loss, mossy fiber sprouting, gliosis, synaptic reorganization, and spontaneous recurrent seizures. Importantly, TPL significantly ameliorates characteristic pathological alterations (including hippocampal neuronal loss and gliosis) and cognitive impairments in the temporal lobe epilepsy (TLE) model. These findings suggest that relying solely on acute seizure models for drug screening may overlook certain potential antiepileptic compounds. Chronic epilepsy is characterized by irreversible structural alterations (e.g., hippocampal sclerosis, synaptic reorganization) and persistent molecular dysfunction (e.g., ion channel dysregulation), whereas acute epilepsy involves transient electrical hyperexcitability without permanent damage. Moreover, chronic cases also exhibit sustained neuroinflammation and glial activation, contrasting with the brief, reactive glial response in acute seizures. These findings suggest that the antiepileptic mechanism of TPL involves neuroprotective effects on the epileptic focus, rather than merely symptomatic seizure control. Collectively, this research provides solid experimental evidence for the preclinical development of TPL as a potential antiepileptic drug.

The chronic KA model effectively mimics recurrent seizure episodes observed in human epilepsy, making it an excellent model for studying the complex cerebral states in chronic epilepsy. EEG is a key diagnostic tool for epileptic disorders. By EEG recording, we found TPL reduced the frequency of interictal spikes and seizures in the KA model by hippocampal EEG recording. No seizure‐like activities were recorded in most of the mice treated with TPL at 10 or 50 μg/kg. On the other hand, psychiatric comorbidities have been considered an integral manifestation of many patients with TLE [36], as also suggested by the International League against Epilepsy [43]. Here, we found TPL also had beneficial effects on the behavioral and cognitive comorbidities in both the novel object recognition test and the location recognition test. These results suggest that a low dose of TPL had a strong antiepileptic effect in the mouse KA model.

The neural circuit dysfunction, especially enhanced excitation and impaired inhibition, is the key aspect of the epileptic hippocampus. Here, we found that TPL improved hippocampal GABAergic inhibition (braking effect) in the epileptic mice in vivo. Besides, we found that TPL also enhanced the expression of VGAT (a vesicular transporter essential for GABAergic neurotransmission) in the DG area in the KA mouse model of TLE. The enhanced fire rate of hippocampal neurons may be the key mechanism underlying both ictal genesis [44] and cognitive impairments [45] in epileptic mice. Both activation of focal interneurons and inhibition of focal pyramidal neurons are antiepileptic and may improve cognitive impairment in epileptic mice [46, 47]. The hippocampal GABAergic inhibition is also the key downstream effector of the antiepileptic entorhinal‐hippocampal circuit that has been confirmed by our previous studies [31]. Recently, Bui et al. found mossy cells, a glutamatergic cell population in the hilus of the dentate gyrus, control both spontaneous convulsive seizures and spatial memory in a well‐established model of TLE with KA injected unilaterally into the left dorsal hippocampus similar to the current study [48]. Of note, most neurons that we recorded are also putative glutamatergic cells in the dentate gyrus in the present study. Thus, these results indicate TPL acts antiepileptic effects by rebalance the excitation and inhibition in the epileptic hippocampus, especially in the dentate gyrus area.

Growing evidence highlights the role of neuroinflammation, particularly the involvement of IL‐1β in the pathogenesis and progression of epilepsy. It has been found that IL‐1β is upregulated in epileptic focal areas and may be primarily expressed in the activated astrocytes in chronic epilepsy situ [49]. More important, recent study has confirmed that IL‐1R1, the receptor of IL‐1β, mainly expressed in the hippocampus of brain [50]. Nemeth DP et al. found that activated astrocytes release excessive IL‐1β in epileptic conditions, which binds to IL‐1R1 abundantly expressed on both glutamatergic and GABAergic neurons [51]. Roseti C et al. found IL‐1β/IL‐1R1 signaling could modulate GABAergic signaling in tissue samples from TLE patients [11]. Furthermore, administration of IL‐1β antagonist in KA mice was found to upregulate expression of GABA transporter types 1 and 3 [52]. Based on bioinformatic analysis, we further found the main cluster of TPL targets, including NLRP3, PTGS2, IL‐1 family, TNF, and some chemokines, where IL‐1β also existed in the main cluster of co‐expression network from human epileptic hippocampus. Consistent with bioinformatic results, we found TPL inhibited astrogliosis and decreased proinflammatory cytokine IL‐1β in the epileptic hippocampus. Thus, these results indicate that TPL modifies the hippocampal IL‐1β‐related inflammatory environment, which is a promising way for epileptic treatment. To further confirm whether IL‐1β is related to the antiepileptic effect of TPL. We found focal injection of IL‐1β reduced the hippocampal GABAergic “braking effect” in normal mice. Taken together, these results indicate that TPL may act focal neural circuit rebalance effect by reducing focal IL‐1β expression.

According to previous studies, L‐1β/IL‐1R1 signaling on microglia, astrocytes, and endothelial cells triggers NF‐κB/MAPK signaling, driving neuroinflammation, BBB disruption, and neurodegeneration [53]; however, L‐1β/IL‐1R1 signaling neuron additionally modulates synaptic plasticity (e.g., enhancing NMDA receptor function, altering ion channels) and circuit activity, influencing memory, mood, and excitability, with dysregulation linked to cognitive and psychiatric disorders [54, 55]. In this study, we found that IL1RA, the antagonist of IL1R1, almost totally restored the hippocampal GABAergic “braking effect”; however, TPCA‐1, a selective inhibitor of the NF‐κB pathway, only partly alleviated IL‐1β‐induced reduction in GABAergic “braking effect”, suggesting neuronal IL‐1R1 and additional targets may also contribute to the antiepileptic effects of TPL. To further explore the antiepileptic mechanisms of TPL, we performed transcriptomic analysis. We preliminarily found that TPL may at least both suppress neuroinflammation (such as downregulating genes related to complement activation and cytokine signaling) and modulate neuronal plasticity (such as upregulating genes related to long‐term depression and calcium signaling). These results support TPL‐mediated rebalancing of epilepsy‐associated neuronal networks and highlight the mechanistic complexity underlying this effect. Further studies are needed to elucidate its precise mechanisms.

Several limitations in this study should be noted as follows: (1) While the kainic acid (KA) model is widely used in TLE research, it may not fully capture the genetic, pathological, and mechanistic aspects of the disease [56, 57]. For example, unlike human TLE, which primarily affects the limbic structures, the KA model often leads to more widespread damage, including lesions outside the hippocampus [58]. Thus, our findings necessitate further validation through high‐quality clinical studies. (2) Side effects, such as renal injury and reproductive concerns, are always an important issue for the clinical use of TPL. Here we preliminarily found 5 treated epileptic mice can still have babies about 4 weeks after 4‐week 50 μg/kg TPL treatment. These results suggest low‐dose of TPL (10–50 μg/kg) may be relatively safe. Of note, because rodents have a specific metabolizing enzyme for TPL that humans do not have, further studies still need to be conducted for the safety aspect of TPL [59].

## 5 Conclusions

In conclusion, our results provide pre‐clinical evidence for the antiepileptic use of a low dose of TPL in a classic KA mouse model of TLE. The mechanism underlying the antiepileptic effect of TPL may be via rescuing the neuroinflammation‐related dysfunction in GABAergic neurons through the astrocyte‐IL‐1β‐GABAergic neuron‐glutamatergic neuron axis in mice. Our results suggest TPL is a candidate compound for epileptic treatment. Further clinical and experimental studies are still warranted, where the side effect of TPL should also be carefully evaluated.

## Author Contributions

Zhenghao Xu and Huawei Zhao designed the study. Haimei Lu, Yijun Luo, Mingxuan Han, Yitian Lan, Wenmi Li, Xiu Yu, Xiaoyu Wang, Rongrong Chen performed all the experiments. In addition, Haimei Lu, Yijun Luo, Mingxuan Han, Huawei Zhao, and Zhenghao Xu analyzed the data and prepared the manuscript. All the authors participated in revising the manuscript and agreed to the final version.

## Ethics Statement

This study was approved by the Institutional Animal Care and Use Committee of Zhejiang Chinese Medical University (Approval No. ZSLL‐2016‐125).

## Conflicts of Interest

The authors declare no conflicts of interest.

## Supporting information


**Figure S1:** cns70586‐sup‐0001‐FigureS1.docx.

**Table S1:** cns70586‐sup‐0002‐TableS1.docx.

**Table S2:** cns70586‐sup‐0002‐TableS2.docx.

## Data Availability

The data that support the findings of this study are available from the corresponding author upon reasonable request.

## References

[1] R. Thijs, D , R. Surges , T. O'Brien, J , R. D. Thijs , T. J. O'Brien , and J. W. Sander , “Epilepsy in Adults,” Lancet 393, no. 10172 (2019): 689–701. 30686584 10.1016/S0140-6736(18)32596-0

[2] A. A. Asadi‐Pooya , F. Brigo , S. Lattanzi , and I. Blumcke , “Adult Epilepsy,” Lancet 402, no. 10399 (2023): 412–424. 37459868 10.1016/S0140-6736(23)01048-6

[3] O. Devinsky , D. C. Hesdorffer , D. J. Thurman , S. Lhatoo , and G. Richerson , “Sudden Unexpected Death in Epilepsy: Epidemiology, Mechanisms, and Prevention,” Lancet Neurology 15, no. 10 (2016): 1075–1088. 27571159 10.1016/S1474-4422(16)30158-2

[4] A. Vezzani , J. French , T. Bartfai , and T. Z. Baram , “The Role of Inflammation in Epilepsy,” Nature Reviews Neurology 7, no. 1 (2011): 31–40. 21135885 10.1038/nrneurol.2010.178 PMC3378051

[5] A. Vezzani , S. Balosso , and T. Ravizza , “Neuroinflammatory Pathways as Treatment Targets and Biomarkers in Epilepsy,” Nature Reviews Neurology 15, no. 8 (2019): 459–472. 31263255 10.1038/s41582-019-0217-x

[6] J. Sui , L. Zhan , S. Ji , et al., “Differential Inflammation Responses Determine the Variable Phenotypes of Epilepsy Induced by GABRG2 Mutations,” CNS Neuroscience & Therapeutics 30, no. 2 (2024): e14583, 10.1111/cns.14583. 38357846 PMC10867793

[7] H. Amhaoul , S. Staelens , and S. Dedeurwaerdere , “Imaging Brain Inflammation in Epilepsy,” Neuroscience 279 (2014): 238–252. 25200114 10.1016/j.neuroscience.2014.08.044

[8] L. Librizzi , F. Noe , A. Vezzani , M. de Curtis , and T. Ravizza , “Seizure‐Induced Brain‐Borne Inflammation Sustains Seizure Recurrence and Blood‐Brain Barrier Damage,” Annals of Neurology 72, no. 1 (2012): 82–90. 22829270 10.1002/ana.23567

[9] T. Ravizza , M. Scheper , R. Di Sapia , J. Gorter , E. Aronica , and A. Vezzani , “mTOR and Neuroinflammation in Epilepsy: Implications for Disease Progression and Treatment,” Nature Reviews Neuroscience 25, no. 5 (2024): 334–350. 38531962 10.1038/s41583-024-00805-1

[10] A. Dey , X. Kang , J. Qiu , Y. Du , and J. Jiang , “Anti‐Inflammatory Small Molecules to Treat Seizures and Epilepsy: From Bench to Bedside,” Trends in Pharmacological Sciences 37, no. 6 (2016): 463–484. 27062228 10.1016/j.tips.2016.03.001 PMC5064857

[11] C. Roseti , E. A. van Vliet , P. Cifelli , et al., “GABAA Currents Are Decreased by IL‐1beta in Epileptogenic Tissue of Patients With Temporal Lobe Epilepsy: Implications for Ictogenesis,” Neurobiology of Disease 82 (2015): 311–320. 26168875 10.1016/j.nbd.2015.07.003

[12] Z. H. Xu , Y. Wang , A. F. Tao , et al., “Interleukin‐1 Receptor Is a Target for Adjunctive Control of Diazepam‐Refractory Status Epilepticus in Mice,” Neuroscience 328 (2016): 22–29. 27133574 10.1016/j.neuroscience.2016.04.036

[13] M. Maroso , S. Balosso , T. Ravizza , et al., “Toll‐Like Receptor 4 and High‐Mobility Group Box‐1 Are Involved in Ictogenesis and Can Be Targeted to Reduce Seizures,” Nature Medicine 16, no. 4 (2010): 413–419. 10.1038/nm.2127 20348922

[14] J. Zhao , Y. Wang , C. Xu , et al., “Therapeutic Potential of an Anti‐High Mobility Group Box‐1 Monoclonal Antibody in Epilepsy,” Brain, Behavior, and Immunity 64 (2017): 308–319. 28167116 10.1016/j.bbi.2017.02.002

[15] M. Zaben , N. Haan , F. Sharouf , et al., “IL‐1beta and HMGB1 Are Anti‐Neurogenic to Endogenous Neural Stem Cells in the Sclerotic Epileptic Human Hippocampus,” Journal of Neuroinflammation 18, no. 1 (2021): 218. 34548070 10.1186/s12974-021-02265-1 PMC8454003

[16] S. Balosso , T. Ravizza , E. Aronica , et al., “The Dual Role of TNF‐Alpha and Its Receptors in Seizures,” Experimental Neurology 247 (2013): 267–271. 23707217 10.1016/j.expneurol.2013.05.010

[17] T. C. Sanchez , R. Spelat , F. Spada , et al., “Exosomal TNF‐Alpha Mediates Voltage‐Gated Na+ Channel 1.6 Overexpression and Contributes to Brain Tumor‐Induced Neuronal Hyperexcitability,” Journal of Clinical Investigation 134, no. 18 (2024). 10.1172/JCI166271 PMC11405049 39088270

[18] P. Noel , D. D. Von Hoff , A. K. Saluja , M. Velagapudi , E. Borazanci , and H. Han , “Triptolide and Its Derivatives as Cancer Therapies,” Trends in Pharmacological Sciences 40, no. 5 (2019): 327–341. 30975442 10.1016/j.tips.2019.03.002

[19] J. Gao , Y. Zhang , X. Liu , X. Wu , L. Huang , and W. Gao , “Triptolide: Pharmacological Spectrum, Biosynthesis, Chemical Synthesis and Derivatives,” Theranostics 11, no. 15 (2021): 7199–7221. 34158845 10.7150/thno.57745 PMC8210588

[20] X. Wang , Y. Zu , L. Huang , et al., “Treatment of Rheumatoid Arthritis With Combination of Methotrexate and *Tripterygium wilfordii*: A Meta‐Analysis,” Life Sciences 171 (2017): 45–50. 28088452 10.1016/j.lfs.2017.01.004

[21] Q. Wang , B. Xiao , S. Cui , et al., “Triptolide Treatment Reduces Alzheimer's Disease (AD)‐Like Pathology Through Inhibition of BACE1 in a Transgenic Mouse Model of AD,” Disease Models & Mechanisms 7, no. 12 (2014): 1385–1395. 25481013 10.1242/dmm.018218 PMC4257007

[22] X. Hu , Y. Dong , X. Jin , et al., “The Novel and Potent Anti‐Depressive Action of Triptolide and Its Influences on Hippocampal Neuroinflammation in a Rat Model of Depression Comorbidity of Chronic Pain,” Brain, Behavior, and Immunity 64 (2017): 180–194. 28300618 10.1016/j.bbi.2017.03.005

[23] J. Wang , Y. Qiao , R. S. Yang , et al., “The Synergistic Effect of Treatment With Triptolide and MK‐801 in the Rat Neuropathic Pain Model,” Molecular Pain 13 (2017): 2071416956. 10.1177/1744806917746564 PMC5734437 29166839

[24] J. M. Li , Y. Zhang , L. Tang , et al., “Effects of Triptolide on Hippocampal Microglial Cells and Astrocytes in the APP/PS1 Double Transgenic Mouse Model of Alzheimer's Disease,” Neural Regeneration Research 11, no. 9 (2016): 1492–1498. 27857756 10.4103/1673-5374.191224 PMC5090855

[25] X. Lu , B. Yang , H. Yu , et al., “Epigenetic Mechanisms Underlying the Effects of Triptolide and Tripchlorolide on the Expression of Neuroligin‐1 in the Hippocampus of APP/PS1 Transgenic Mice,” Pharmaceutical Biology 57, no. 1 (2019): 453–459. 31311385 10.1080/13880209.2019.1629463 PMC6691810

[26] Y. S. Wan , Y. You , Q. Y. Ding , et al., “Triptolide Protects Against White Matter Injury Induced by Chronic Cerebral Hypoperfusion in Mice,” Acta Pharmacologica Sinica 43, no. 1 (2022): 15–25. 33824460 10.1038/s41401-021-00637-0 PMC8724323

[27] A. F. Tao , Z. H. Xu , B. Chen , et al., “The Pro‐Inflammatory Cytokine Interleukin‐1beta Is a Key Regulatory Factor for the Postictal Suppression in Mice,” CNS Neuroscience & Therapeutics 21, no. 8 (2015): 642–650. 26096304 10.1111/cns.12416 PMC6495150

[28] W. W. Hu , Q. Fang , Z. H. Xu , et al., “Chronic h1‐Antihistamine Treatment Increases Seizure Susceptibility After Withdrawal by Impairing Glutamine Synthetase,” CNS Neuroscience & Therapeutics 18, no. 8 (2012): 683–690. 22742831 10.1111/j.1755-5949.2012.00356.x PMC6493495

[29] Y. J. Dai , Z. H. Xu , B. Feng , et al., “Gender Difference in Acquired Seizure Susceptibility in Adult Rats After Early Complex Febrile Seizures,” Neuroscience Bulletin 30, no. 6 (2014): 913–922. 25394585 10.1007/s12264-014-1482-8 PMC5562567

[30] Y. P. Jiang , Y. Jin , J. Bao , et al., “Inconsistent Time‐Dependent Effects of Tetramethylpyrazine on Primary Neurological Disorders and Psychiatric Comorbidities,” Frontiers in Pharmacology 12 (2021): 708517. 34489702 10.3389/fphar.2021.708517 PMC8417558

[31] Z. Xu , Y. Wang , B. Chen , et al., “Entorhinal Principal Neurons Mediate Brain‐Stimulation Treatments for Epilepsy,” eBioMedicine 14 (2016): 148–160. 27908611 10.1016/j.ebiom.2016.11.027 PMC5161446

[32] Y. Wang , C. Xu , Z. Xu , et al., “Depolarized GABAergic Signaling in Subicular Microcircuits Mediates Generalized Seizure in Temporal Lobe Epilepsy,” Neuron 95, no. 1 (2017): 92–105. 28648501 10.1016/j.neuron.2017.06.004

[33] Y. Wang , Y. Wang , C. Xu , et al., “Direct Septum‐Hippocampus Cholinergic Circuit Attenuates Seizure Through Driving Somatostatin Inhibition,” Biological Psychiatry 87, no. 9 (2020): 843–856. 31987494 10.1016/j.biopsych.2019.11.014

[34] F. Lu , H. Lu , M. Xie , et al., “Limited Preventive Effect of Prednisone on Neuropsychiatric Symptoms in Murine Systemic Lupus Erythematosus,” Inflammopharmacology 27, no. 3 (2019): 511–520. 30911862 10.1007/s10787-019-00587-4

[35] H. Lu , M. Xie , S. Li , et al., “Modification of 6 Hz Corneal Kindled Mouse Model for Drug‐Resistant Epilepsy and Its Treatment by Three Traditional Chinese Medicine Prescriptions,” Acta Pharmaceutica Sinica (Chinese) 53, no. 7 (2018): 1048–1053.

[36] J. Royer , S. Lariviere , R. Rodriguez‐Cruces , et al., “Cortical Microstructural Gradients Capture Memory Network Reorganization in Temporal Lobe Epilepsy,” Brain 146, no. 9 (2023): 3923–3937. 37082950 10.1093/brain/awad125 PMC10473569

[37] A. E. Tipton , D. A. Y. Cruz , K. Hixson , et al., “Selective Neuronal Knockout of STAT3 Function Inhibits Epilepsy Progression, Improves Cognition, and Restores Dysregulated Gene Networks in a Temporal Lobe Epilepsy Model,” Annals of Neurology 94, no. 1 (2023): 106–122. 36935347 10.1002/ana.26644 PMC10313781

[38] M. R. Johnson , J. Behmoaras , L. Bottolo , et al., “Systems Genetics Identifies Sestrin 3 as a Regulator of a Proconvulsant Gene Network in Human Epileptic Hippocampus,” Nature Communications 6 (2015): 6031. 10.1038/ncomms7031 PMC4627576 25615886

[39] Z. Sun , M. Du , Y. Lu , and C. Q. Zeng , “Effects of Triptolide on the Expression of MHC II in Microglia in Kainic Acid‐Induced Epilepsy,” Molecular Medicine Reports 17, no. 6 (2018): 8357–8362. 29693706 10.3892/mmr.2018.8891

[40] X. Pan , S. F. Zou , C. X. Zeng , et al., “Effects of Tripterolide on kv1.1 Expression of Voltage Gated Potassium Channels in Epileptic Rats,” Journal of NeuroInterventional Surgery (Chinese) 39, no. 2 (2012): 108–113.

[41] Y. Cui , X. Jiang , and J. Feng , “The Therapeutic Potential of Triptolide and Celastrol in Neurological Diseases,” Frontiers in Pharmacology 13 (2022): 1024955. 36339550 10.3389/fphar.2022.1024955 PMC9626530

[42] K. Leclercq , A. Matagne , and R. M. Kaminski , “Low Potency and Limited Efficacy of Antiepileptic Drugs in the Mouse 6 Hz Corneal Kindling Model,” Epilepsy Research 108, no. 4 (2014): 675–683. 24661426 10.1016/j.eplepsyres.2014.02.013

[43] M. Gandy , R. Michaelis , J. Acraman , et al., “Integrated Psychological Care Services Within Seizure Settings: Key Components and Implementation Factors Among Example Services in Four ILAE Regions: A Report by the ILAE Psychiatry Commission,” Epilepsia 64, no. 7 (2023): 1766–1784. 37227085 10.1111/epi.17647

[44] B. Styr , N. Gonen , D. Zarhin , et al., “Mitochondrial Regulation of the Hippocampal Firing Rate Set Point and Seizure Susceptibility,” Neuron 102, no. 5 (2019): 1009–1024. 31047779 10.1016/j.neuron.2019.03.045 PMC6559804

[45] J. B. Kahn , R. G. Port , C. Yue , H. Takano , and D. A. Coulter , “Circuit‐Based Interventions in the Dentate Gyrus Rescue Epilepsy‐Associated Cognitive Dysfunction,” Brain 142, no. 9 (2019): 2705–2721. 31363737 10.1093/brain/awz209 PMC6736326

[46] E. Krook‐Magnuson , C. Armstrong , M. Oijala , and I. Soltesz , “On‐Demand Optogenetic Control of Spontaneous Seizures in Temporal Lobe Epilepsy,” Nature Communications 4 (2013): 1376. 10.1038/ncomms2376 PMC3562457 23340416

[47] Y. Wang , J. Liang , L. Chen , et al., “Pharmaco‐Genetic Therapeutics Targeting Parvalbumin Neurons Attenuate Temporal Lobe Epilepsy,” Neurobiology of Disease 117 (2018): 149–160. 29894753 10.1016/j.nbd.2018.06.006

[48] A. D. Bui , T. M. Nguyen , C. Limouse , et al., “Dentate Gyrus Mossy Cells Control Spontaneous Convulsive Seizures and Spatial Memory,” Science 359, no. 6377 (2018): 787–790. 29449490 10.1126/science.aan4074 PMC6040648

[49] T. Ravizza , F. Noe , D. Zardoni , V. Vaghi , M. Sifringer , and A. Vezzani , “Interleukin Converting Enzyme Inhibition Impairs Kindling Epileptogenesis in Rats by Blocking Astrocytic IL‐1beta Production,” Neurobiology of Disease 31, no. 3 (2008): 327–333. 18632279 10.1016/j.nbd.2008.05.007

[50] D. J. Disabato , D. P. Nemeth , X. Liu , et al., “Interleukin‐1 Receptor on Hippocampal Neurons Drives Social Withdrawal and Cognitive Deficits After Chronic Social Stress,” Molecular Psychiatry 26, no. 9 (2021): 4770–4782. 32444870 10.1038/s41380-020-0788-3 PMC8730339

[51] D. P. Nemeth , X. Liu , M. C. Monet , et al., “Localization of Brain Neuronal IL‐1R1 Reveals Specific Neural Circuitries Responsive to Immune Signaling,” Journal of Neuroinflammation 21, no. 1 (2024): 303. 39563437 10.1186/s12974-024-03287-1 PMC11575132

[52] J. Su , J. Yin , W. Qin , S. Sha , J. Xu , and C. Jiang , “Role for Pro‐Inflammatory Cytokines in Regulating Expression of GABA Transporter Type 1 and 3 in Specific Brain Regions of Kainic Acid‐Induced Status Epilepticus,” Neurochemical Research 40, no. 3 (2015): 621–627. 25708016 10.1007/s11064-014-1504-y

[53] S. M. Krasnow , J. G. Knoll , S. C. Verghese , et al., “Amplification and Propagation of Interleukin‐1beta Signaling by Murine Brain Endothelial and Glial Cells,” Journal of Neuroinflammation 14, no. 1 (2017): 133. 28668091 10.1186/s12974-017-0908-4 PMC5494131

[54] B. Viviani , S. Bartesaghi , F. Gardoni , et al., “Interleukin‐1beta Enhances NMDA Receptor‐Mediated Intracellular Calcium Increase Through Activation of the Src Family of Kinases,” Journal of Neuroscience 23, no. 25 (2003): 8692–8700. 14507968 10.1523/JNEUROSCI.23-25-08692.2003 PMC6740426

[55] F. Zipp , S. Bittner , and D. P. Schafer , “Cytokines as Emerging Regulators of Central Nervous System Synapses,” Immunity 56, no. 5 (2023): 914–925. 37163992 10.1016/j.immuni.2023.04.011 PMC10233069

[56] P. A. Williams , J. L. Hellier , A. M. White , et al., “Development of Spontaneous Seizures After Experimental Status Epilepticus: Implications for Understanding Epileptogenesis,” Epilepsia 48, no. Suppl 5 (2007): 157–163. 17910596 10.1111/j.1528-1167.2007.01304.x

[57] J. Wang , W. Wu , J. Wan , et al., “Preliminary Study on the Mechanism of SAHA in the Treatment of Refractory Epilepsy Induced by GABRG2(F343L) Mutation,” Biochemical Pharmacology 227 (2024): 116449, 10.1016/j.bcp.2024.116449. 39053637

[58] V. Bouilleret , A. Nehlig , C. Marescaux , and I. J. Namer , “Magnetic Resonance Imaging Follow‐Up of Progressive Hippocampal Changes in a Mouse Model of Mesial Temporal Lobe Epilepsy,” Epilepsia 41, no. 6 (2000): 642–650. 10840394 10.1111/j.1528-1157.2000.tb00223.x

[59] T. Wu , H. Yang , M. Yuan , et al., “Comparative Study of Metabolic Clearance and Enzymatic Kinetics of Triptolide in Human and Rat Liver Microsomes,” Chinese Pharmacological Bulletin (Chinese) 34, no. 10 (2018): 1414–1419.

