# Supplementary material for: Triptolide Inhibits Epileptic Seizures by Rescuing the Neuroinflammation‐Related GABAergic Dysfunction in Mice
Source: CNS Neurosci Ther. 2025 Aug 29;31(8):e70586. doi: 10.1111/cns.70586 (PMC12394891; doi:10.1111/cns.70586)
Supplement: Supplementary file 1 — Figure S1: cns70586‐sup‐0001‐FigureS1.docx. [file CNS-31-e70586-s002.docx]

**Supplementary document 1**

**Original uncropped Western blot images**


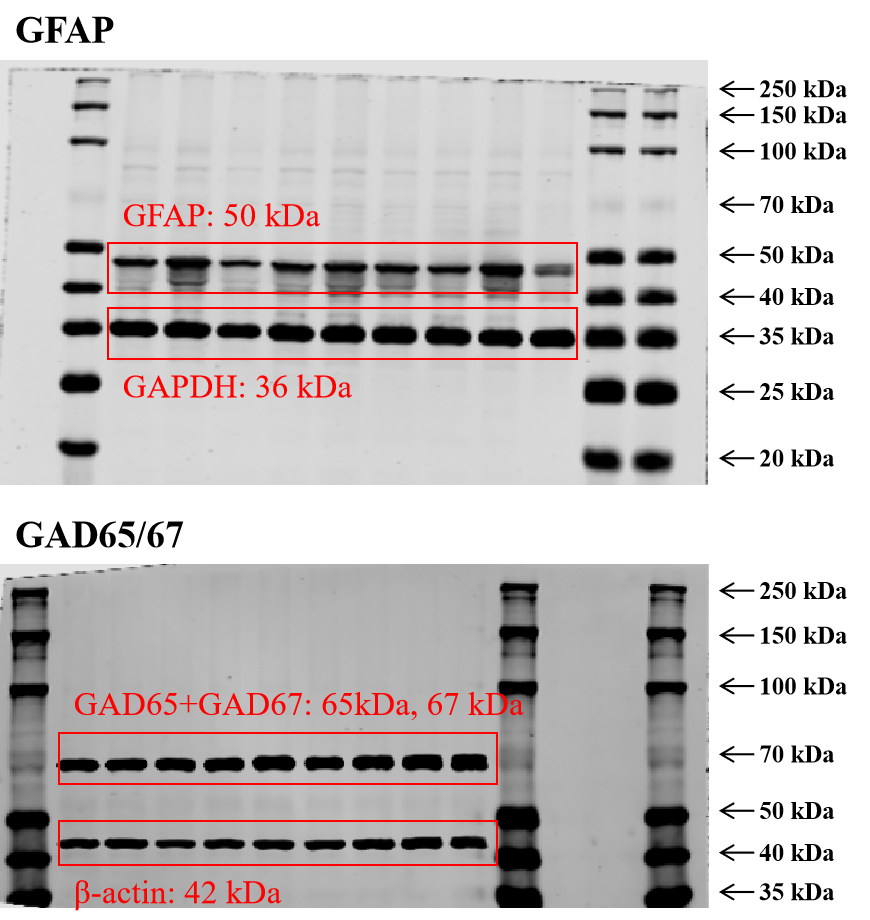


**Figure S1. Original uncropped Western blot images for Figure 6**
