# Supplementary material for: Triptolide Inhibits Epileptic Seizures by Rescuing the Neuroinflammation‐Related GABAergic Dysfunction in Mice
Source: CNS Neurosci Ther. 2025 Aug 29;31(8):e70586. doi: 10.1111/cns.70586 (PMC12394891; doi:10.1111/cns.70586)
Supplement: Supplementary file 2 — Table S1: cns70586‐sup‐0002‐TableS1.docx. [file CNS-31-e70586-s001.docx]

**Supplementary Table 1. Details about statistical tests**

| **Figures** | **Method** | **t, F, df value** | **P value** | **Post hoc method** | **Normality test**  **(Method, p value)** | **Homogeneity of variance test**  **(Method, p value)** |
| --- | --- | --- | --- | --- | --- | --- |
| Figure 1B | Kruskal-Wallis test | Kruskal-Wallis statistic=9.563 | P=0.0147 | Dunn’s | / | / |
| Figure 1C | Student's t-test | t=0.9535, df=10 | P=0.3628 | NA | Shaprio-Wilk test, P=0.4733, P=0.0932 | F test, P=0.5540 |
| Figure 1D | Kruskal-Wallis test | Kruskal-Wallis statistic=13.64 | P=0.0010 | Dunn’s | / | / |
| Figure 1E | Student's t-test | t=0.7276, df=10 | P=0.4835 | NA | Shaprio-Wilk test, P=0.4207, P=0.4150 | F test, P=0.9401 |
| Figure 2B | Two-way ANOVA | F (1, 23) =6.367 | P=0.0190 | LSD | Shaprio-Wilk test, P=0.7507, P=0.7968 | / |
| Figure 2C | Log-rank (Mantel-Cox) test | Chi square = 2.781, df = 1 | P=0.0954 | NA | / | / |
| Figure 2D | Student's t-test  Student's t-test | t=1.754, df=23;  t=1.754, df=23 | P=0.0928;  P=0.0928 | NA  NA | Shaprio-Wilk test, P=0.2795, P=0.1159;  Shaprio-Wilk test, P=0.2795, P=0.1159 | F test, P=0.6531;  F test, P=0.6531 |
| Figure 2E | Mann Whitney test  Student's t-test | Mann-Whitney U=51.50;  t=0.6683, df=23 | P=0.1532;  P=0.5106 | NA  NA | Shaprio-Wilk test, P=0.2437, P=0.0216;  Shaprio-Wilk test, P=0.6749, P=0.1036; | F test, P=0.8756;  F test, P=0.1629 |
| Figure 3B | Mann Whitney test  Mann Whitney test | Mann-Whitney U=17.50;  Mann-Whitney U=3.50 | P>0.9999;  P=0.0152 | NA  NA | Shaprio-Wilk test, P<0.0001, P<0.0001;  Shaprio-Wilk test, P<0.0001, P=0.0359 | F test, P=0.1544;  F test, P=0.0492 |
| Figure 3C | Fisher's exact test | NA | P=0.0152 | NA | / | / |
| Figure 3D | Mann Whitney test | Mann-Whitney U=3 | P=0.0195 | NA | Shaprio-Wilk test, P=0.0065, P=0.2117 | F test, P=0.2376 |
| Figure 4B | One-way ANOVA | F (3, 23) = 4.373 | P=0.0141 | Tukey | Shaprio-Wilk test, P=0.3087, P=0.3920, P=0.6253, P=0.8160 | Brown-Forsythe test, P=0.2458 |
| Figure 4C | Kruskal-Wallis test | Kruskal-Wallis statistic=8.649 | P=0.0343 | Dunn’s | Shaprio-Wilk test, P=0.6723, P=0.1902, P<0.0001, P<0.0001 | Brown-Forsythe test, P=0.0431 |
| Figure 4E | Kruskal-Wallis test | Kruskal-Wallis statistic=18.46 | P=0.0004 | Dunn’s | Shaprio-Wilk test, P=0.1755, P=0.2581, P=0.0451, P=0.0493 | Brown-Forsythe test, P=0.4205 |
| Figure 4F | One-way ANOVA | F (3, 55) = 3.001 | P=0.0382 | Tukey | Shaprio-Wilk test, P=0.5287, P=0.4647, P=0.4468, P=0.3471 | Brown-Forsythe test, P=0.2437 |
| Figure 4H | Student's t-test | t=4.424, df=8 | P=0.0022 | NA | Shaprio-Wilk test, P=0.8046, P=0.7678 | F test, P=0.4089 |
| Figure 4I | Student's t-test | t=12.44, df=8 | P<0.0001 | NA | Shaprio-Wilk test, P=0.8140, P=0.4677 | F test, P=0.2564 |
| Figure 5D | Kruskal-Wallis test | Kruskal-Wallis statistic=20.55 | P<0.0001 | Dunn’s | Shaprio-Wilk test, P=0.0047, P=0.0057, P=0.0023 | Brown-Forsythe test, P=0.1023 |
| Figure 5F | Kruskal-Wallis test;  Kruskal-Wallis test | Kruskal-Wallis statistic=5.689;  Kruskal-Wallis statistic=2.222; | P=0.0286;  P=0.3821 | Dunn’s;  Dunn’s; | Shaprio-Wilk test, P=0.0448, P=0.6470, P=0.1101;  Shaprio-Wilk test, P=0.3267, P=0.0420, P=0.0408; | Brown-Forsythe test, P=0.9796;  Brown-Forsythe test, P=0.9824; |
| Figure 5G | One-way ANOVA;  One-way ANOVA | F (2, 6) = 7.069；  F (2, 6) = 4.922 | P=0.0264；  P=0.0543 | Tukey  Tukey | Shaprio-Wilk test, P=0.9475, P=0.8168, P=0.1223;  Shaprio-Wilk test, P=0.1321, P=0.3976, P=0.2351 | Brown-Forsythe test, P=0.8100；Brown-Forsythe test, P=0.8590 |
| Figure 5H | One-way ANOVA;  One-way ANOVA | F (2, 6) = 4.132  F (2, 6) = 7.473 | P=0.0744  P=0.0235 | Tukey  Tukey | Shaprio-Wilk test, P=0.2333, P=0.1719, P=0.5087;  Shaprio-Wilk test, P=0.6489, P=0.8551, P=0.3218 | Brown-Forsythe test, P=0.9213；Brown-Forsythe test, P=0.8590 |
| Figure 6A | One-way ANOVA | F (2, 6) = 7.744 | P=0.0218 | Tukey | Shaprio-Wilk test, P=0.8404, P=0.5702, P=0.2286 | Brown-Forsythe test, P=0.4228 |
| Figure 6B | One-way ANOVA | F (2, 6) = 0.6737 | P=0.5446 | Tukey | Shaprio-Wilk test, P=0.0899, P=0.5150, P=0.5999 | Brown-Forsythe test, P=0.5129 |
| Figure 6D | One-way ANOVA | F (2, 13) = 29.47 | P＜0.0001 | Tukey | Shaprio-Wilk test, P=0.5768, P=0.5225, P=0.3879 | Brown-Forsythe test, P=0.5957 |
| Figure 6E | One-way ANOVA | F (2, 13) = 6.888 | P=0.0091 | Tukey | Shaprio-Wilk test, P=0.1383, P=0.2347, P=0.2413 | Brown-Forsythe test, P=0.4080 |
| Figure 6F | One-way ANOVA | F (2, 13) = 14.56 | P=0.0005 | Tukey | Shaprio-Wilk test, P=0.8380, P=0.2724, P=0.2286 | Brown-Forsythe test, P=0.5200 |
| Figure 7C | Mann Whitney test | Mann-Whitney U=0 | P=0.0286 | NA | Shaprio-Wilk test, P=0.8947, P=0.0293 | F test, P=0.1411 |
| Figure 7F | Kruskal-Wallis test | Kruskal-Wallis statistic=16.76 | P=0.0008 | Uncorrected Dunn’s | Shaprio-Wilk test, P=0.8654, P=0.0001, P=0.3197, P=0.2605 | Brown-Forsythe test, P=0.7181 |
